# Convergent Degenerated Regulatory Elements Associated with Limb Loss in Limbless Amphibians and Reptiles

**DOI:** 10.1093/molbev/msae239

**Published:** 2024-11-12

**Authors:** Chenglong Zhu, Shengyou Li, Daizhen Zhang, Jinjin Zhang, Gang Wang, Botong Zhou, Jiangmin Zheng, Wenjie Xu, Zhengfei Wang, Xueli Gao, Qiuning Liu, Tingfeng Xue, Huabin Zhang, Chunhui Li, Baoming Ge, Yuxuan Liu, Qiang Qiu, Huixian Zhang, Jinghui Huang, Boping Tang, Kun Wang

**Affiliations:** Shaanxi Key Laboratory of Qinling Ecological Intelligent Monitoring and Protection, School of Ecology and Environment, Northwestern Polytechnical University, Xi’an 710072, China; Department of Orthopaedics, Xijing Hospital, The Fourth Military Medical University, Xi’an 710032, China; Jiangsu Key Laboratory for Bioresources of Saline Soils, Jiangsu Provincial Key Laboratory of Coastal Wetland Bioresources and Environmental Protection, Jiangsu Synthetic Innovation Center for Coastal Bio-agriculture, Yancheng Teachers University, Yancheng City 224002, China; State Key Laboratory of Genetic Resources and Evolution, Kunming Natural History Museum of Zoology, Kunming Institute of Zoology, Chinese Academy of Sciences, Kunming, Yunnan, 650223, China; Jiangsu Key Laboratory for Bioresources of Saline Soils, Jiangsu Provincial Key Laboratory of Coastal Wetland Bioresources and Environmental Protection, Jiangsu Synthetic Innovation Center for Coastal Bio-agriculture, Yancheng Teachers University, Yancheng City 224002, China; Shaanxi Key Laboratory of Qinling Ecological Intelligent Monitoring and Protection, School of Ecology and Environment, Northwestern Polytechnical University, Xi’an 710072, China; Shaanxi Key Laboratory of Qinling Ecological Intelligent Monitoring and Protection, School of Ecology and Environment, Northwestern Polytechnical University, Xi’an 710072, China; Shaanxi Key Laboratory of Qinling Ecological Intelligent Monitoring and Protection, School of Ecology and Environment, Northwestern Polytechnical University, Xi’an 710072, China; Jiangsu Key Laboratory for Bioresources of Saline Soils, Jiangsu Provincial Key Laboratory of Coastal Wetland Bioresources and Environmental Protection, Jiangsu Synthetic Innovation Center for Coastal Bio-agriculture, Yancheng Teachers University, Yancheng City 224002, China; Shaanxi Key Laboratory of Qinling Ecological Intelligent Monitoring and Protection, School of Ecology and Environment, Northwestern Polytechnical University, Xi’an 710072, China; Jiangsu Key Laboratory for Bioresources of Saline Soils, Jiangsu Provincial Key Laboratory of Coastal Wetland Bioresources and Environmental Protection, Jiangsu Synthetic Innovation Center for Coastal Bio-agriculture, Yancheng Teachers University, Yancheng City 224002, China; Shaanxi Key Laboratory of Qinling Ecological Intelligent Monitoring and Protection, School of Ecology and Environment, Northwestern Polytechnical University, Xi’an 710072, China; Jiangsu Key Laboratory for Bioresources of Saline Soils, Jiangsu Provincial Key Laboratory of Coastal Wetland Bioresources and Environmental Protection, Jiangsu Synthetic Innovation Center for Coastal Bio-agriculture, Yancheng Teachers University, Yancheng City 224002, China; Shaanxi Key Laboratory of Qinling Ecological Intelligent Monitoring and Protection, School of Ecology and Environment, Northwestern Polytechnical University, Xi’an 710072, China; Jiangsu Key Laboratory for Bioresources of Saline Soils, Jiangsu Provincial Key Laboratory of Coastal Wetland Bioresources and Environmental Protection, Jiangsu Synthetic Innovation Center for Coastal Bio-agriculture, Yancheng Teachers University, Yancheng City 224002, China; Shaanxi Key Laboratory of Qinling Ecological Intelligent Monitoring and Protection, School of Ecology and Environment, Northwestern Polytechnical University, Xi’an 710072, China; Shaanxi Key Laboratory of Qinling Ecological Intelligent Monitoring and Protection, School of Ecology and Environment, Northwestern Polytechnical University, Xi’an 710072, China; CAS Key Laboratory of Tropical Marine Bio-Resources and Ecology, Guangdong Provincial Key Laboratory of Applied Marine Biology, South China Sea Institute of Oceanology, Chinese Academy of Sciences, Guangzhou 510301, China; Department of Orthopaedics, Xijing Hospital, The Fourth Military Medical University, Xi’an 710032, China; Jiangsu Key Laboratory for Bioresources of Saline Soils, Jiangsu Provincial Key Laboratory of Coastal Wetland Bioresources and Environmental Protection, Jiangsu Synthetic Innovation Center for Coastal Bio-agriculture, Yancheng Teachers University, Yancheng City 224002, China; Shaanxi Key Laboratory of Qinling Ecological Intelligent Monitoring and Protection, School of Ecology and Environment, Northwestern Polytechnical University, Xi’an 710072, China

**Keywords:** banna caecilian, adaptive evolution, limbless

## Abstract

Limbs are a defining characteristic of tetrapods, yet numerous taxa, primarily among amphibians and reptiles, have independently lost limbs as an adaptation to new ecological niches. To elucidate the genetic factors contributing to this convergent limb loss, we present a 12 Gb chromosome-level assembly of the Banna caecilian (*Ichthyophis bannanicus*), a limbless amphibian. Our comparative analysis, which includes the reconstruction of amphibian karyotype evolution, reveals constrained gene length evolution in a subset of developmental genes across 3 large genomes. Investigation of limb development genes uncovered the loss of *Grem1* in caecilians and *Tulp3* in snakes. Interestingly, caecilians and snakes share a significantly larger number of convergent degenerated conserved noncoding elements than limbless lizards, which have a shorter evolutionary history of limb loss. These convergent degenerated conserved noncoding elements overlap significantly with active genomic regions during mouse limb development and are conserved in limbed species, suggesting their essential role in limb patterning in the tetrapod common ancestor. While most convergent degenerated conserved noncoding elements emerged in the jawed vertebrate ancestor, coinciding with the origin of paired appendage, more recent degenerated conserved noncoding elements also contribute to limb development, as demonstrated through functional experiments. Our study provides novel insights into the regulatory elements associated with limb development and loss, offering an evolutionary perspective on the genetic basis of morphological specialization.

## Introduction

Limbs have been a crucial factor in the terrestrial success of tetrapods (Ashley-Ross et al. 2013), enabling them to adapt to various ecological niches. However, some tetrapod taxa have evolved to occupy new niches by abandoning their limbs. These limbless tetrapods include snakes, limbless lizards, and an extant order of limbless amphibians known as caecilians (order Gymnophiona). With over 222 species (https://amphibiaweb.org, accessed 16 Sep 2024), caecilians inhabit damp, tropical regions such as Southeast Asia, selected areas of East and West Africa, and Central and Northern America (Wilkinson et al. 2011). These species show pronounced adaptations for a burrowing lifestyle, including reduced or nonfunctional eyes and heightened reliance on olfactory and tactile senses (Himstedt and Simon 1995; Malonza 2016). These features distinguish them as a distinct group within limbless tetrapods.

Developmental biologists have revealed that limb development is a complex process involving the coordinated action of multiple genes and signaling pathways (Petit et al. 2017; Zhu and Tabin 2023). The apical ectodermal ridge (AER) and the zone of polarizing activity (ZPA) play crucial roles in directing limb outgrowth and patterning, with the AER secreting fibroblast growth factors (FGFs) and the ZPA secreting Sonic hedgehog (SHH) (Zhu and Tabin 2023). Other important genes, such as *Hox* genes and the WNT and BMP signaling pathways, also contribute to the regulation of limb growth and patterning (Geetha-Loganathan et al. 2008; Petit et al. 2017; Zhu and Tabin 2023). The intricate interaction between these genes and signaling pathways directs the development of limb structures. However, our understanding of the regulatory elements involved in tetrapod limb development remains limited, with most of the available research focusing on mouse limb development, particularly from the ENCODE project (Luo et al. 2020).

While these data are essential, it remains unclear whether they are applicable to all tetrapods. Research on limbless tetrapods offers new perspectives on this issue. The most famous case is the ZPA regulatory sequence (ZRS), a long-range enhancer located approximately 1 Mb upstream from its target gene, *Shh*. The ZRS serves as the primary enhancer regulating *Shh* expression in the developing limb bud (Lettice et al. 2002). Interestingly, this sequence has a 17 bp deletion in snakes and is completely lost in caecilians (Kvon et al. 2016; Ovchinnikov et al. 2023). The knock-in of ZRS sequences from the Burmese python (*Python molurus bivittatus*) into mice (FVB strain) resulted in a “serpentized” phenotype, while this deficiency could be rescued with the reintroduction of the missing 17 bp sequence (Kvon et al. 2016). Nevertheless, a recent study indicated that there are only a few conserved regulatory elements that have undergone convergent alternations between limbless lizards and snakes, suggesting that different groups may have distinct mechanisms for limb loss (Roscito et al. 2022). It is worth noting that limb loss in the studied limbless lizards occurred relatively recently, around 40 million years ago (Mya) (Roscito et al. 2022). To gain a deeper understanding of limb-related genomic elements from an evolutionary perspective, it is valuable to conduct in-depth analyses using caecilians, which, like snakes, represent an independent lineage that has undergone limb loss over an extended evolutionary period (caecilians: ∼190 Mya, Kligman et al. 2023; snakes: ∼170 Mya, Garberoglio et al. 2019).

In this study, we generated a chromosome-level genome assembly of the Banna caecilian, which, at 12 Gb, is one of the largest tetrapod genome assemblies, surpassed only by the axolotl (*Ambystoma mexicanum*) (Nowoshilow et al. 2018; Schloissnig et al. 2021). Our analyses revealed independent genome expansion in this species, but dozens of developmental genes showed no significant intron length expansion, unlike the typical pattern seen in most genes of large-genome vertebrates. We further investigated the loss of limb development-related genes and the degeneration of regulatory elements in the Banna caecilian and other limbless tetrapods. Combined with functional experiments, our results revealed that the regulatory elements involved in limb development exhibit complex function and show clear signs of degeneration following limb loss.

## Materials and Methods

### Ethics Approval

All experimental protocols in this study were approved by the Northwestern Polytechnic University Ethics Committee Institutional Review Board (202301024 and 202301143). Every effort was made to minimize animal suffering. All experiments were conducted in accordance with relevant guidelines and regulations.

### Sample Collection and Sequencing

We collected 2 Banna caecilian samples from Xishuangbanna Dai Autonomous Prefecture, Yunnan Province, in 2018. The muscle tissue of the first sample, named “Banna01,” was used for whole-genome sequencing. For PacBio long reads, we used the PacBio Sequel II platform, while for short reads, we used the MGISEQ-2000 platform. To obtain a chromosome-level genome assembly, we performed Hi-C sequencing on the muscle tissue of the second sample, named “Banna02.” Additionally, we conducted transcriptome sequencing on brain, gallbladder, heart, intestine, liver, lung, and skin tissue from “Banna01,” as well as brain, gonad, heart, intestine, kidney, liver, mandible, lung, muscle, skin, spinal cord, spine, testis, and jaw tissues from “Banna02” using the MGISEQ-2000 platform.

### Genome Assembly and Assessment

We used fastp v0.20.0 software (Chen et al. 2018) for quality control of the short reads, filtering out low-quality data with the parameters “-n 0.” To predict the genome size of the Banna caecilian, we used the *k*-mer based method with filtered short reads by using kmerfreq (parameter: -k 27) in SOAPec v2.0 (Luo et al. 2012). NextDenovo v2.3.0 software (Hu et al. 2024) was used to perform genome assembly of the Banna caecilian with PacBio long reads and default parameters. We then used NextPolish v1.3.0 software (Hu et al. 2020) to perform genome correction (parameter: task = best) on the NextDenovo assembly results in combination with filtered short reads. To obtain a chromosome-level genome assembly, we used ALLHiC v0.9.13 software (Zhang et al. 2019) to further scaffold the genome assembly with 21 chromosomes as a group number. The final assembly results were manually corrected using Juicebox Assembly Tools (Durand et al. 2016) and visualized using the plotHicGenome (v0.1.0, https://github.com/Atvar2/plotHicGenome) with default parameters.

To evaluate the assembly results, we aligned 481 UCEs (Kent 2002) using the blat v35 software with sensitive parameters “-minIdentity = 60 -minScore = 30 -minMatch = 1 stepSize = 8 -mask = lower” (Nowoshilow et al. 2018). Alignment lengths of at least 50 base pairs, and representing 75% of the query sequence, were considered to indicate the presence of UCEs in the genomes. We also aligned UCEs to the genomes of the Gaboon caecilian, tiny Cayenne caecilian, and two-lined caecilian for comparison. We used bwa-mem2 v2.2.1 software (Md et al. 2019) with default parameters to align short reads and minimap2 (version: 2.17-r943-dirty) software (Li 2018) with default parameters to align PacBio long reads to the genome to assess the integrity and accuracy of the genome assembly. Finally, we performed a correlation analysis between the physical relative lengths of the Banna caecilian chromosomes extracted from a previously published article (Wen and Pang 1990) and the assembly results to assess the reliability of the chromosome-level assembly.

### Annotation of Banna Caecilian Genome Sequences

We performed repeat sequence annotation on the genome using a combined approach of *de novo* and homology-based annotation. First, we used Tandem Repeats Finder v4.0.9 software (Benson 1999) to annotate simple tandem repeats (parameters: 2 5 7 80 10 50 2000 -d -h -ngs). We then used RepeatModeler v1.0.11 (Saha et al. 2008) to construct a *de novo* TE database, followed by annotation of repetitive sequences using RepeatMasker v4.0.7 software (Tarailo-Graovac and Chen 2009) with default parameters. Simultaneously, homology-based annotation was performed using RepeatMasker v4.0.7 (Tarailo-Graovac and Chen 2009) (parameters: -nolow -norna -no_is -gff) and the RepeatProteinMask tool from the RepeatMasker software package (Tarailo-Graovac and Chen 2009) to align the genome to known repetitive sequence libraries (parameters: -engine ncbi -noLowSimple -pvalue 1e-04). The divergence time of TEs was calculated using the parseRM.pl script (https://github.com/4ureliek/Parsing-RepeatMasker-Outputs). Finally, we integrated all the annotation results and used Bedtools v2.29.2 software (Quinlan and Hall 2010) to convert the sequences corresponding to repetitive elements in the genome to lowercase letters to obtain soft-masked sequences (parameter: maskFastaFromBed -soft).

To predict protein-coding genes, we used a combination of *de novo* annotation and homology-based annotation. First, we performed assembly on transcriptome data from 21 samples across 15 tissues from 2 individuals to obtain a transcriptional protein data set for the Banna caecilian. We used fastp v0.20.0 (Chen et al. 2018) to filter out low-quality bases from the raw data using default parameters. We then performed *de novo* assembly of the transcriptome using SPAdes v3.14.1 software (Bushmanova et al. 2019) and then used TransDecoder v5.5.0 software (Haas and Papanicolaou 2017) to identify and translate the coding regions of the assembled transcript sequences to obtain the protein sequences in the transcriptome assembly. We filtered out protein sequences with less than 30 amino acids and removed redundant sequences using CD-HIT v4.8.1 software (Fu et al. 2012) with default parameters.

Subsequently, we initially aligned the transcriptome protein data and homologous protein sets from chicken, human, mouse, coelacanth, western clawed frog, and two-lined caecilian (supplementary table S15, Supplementary Material online) to the Banna caecilian genome using the blat software with default parameters. Alignments shorter than 70% of the query sequence were filtered out. Next, we clustered the alignment results based on the genomic position information. Here, we retain 3 categories of genomic position information for subsequent annotation: (i) positions supported by homologous proteins in at least 3 different species, (ii) positions supported by evidence from at least 3 different tissue samples in the transcriptome, and (iii) positions supported by at least 1 homologous protein and 1 transcriptomic evidence. Then, we used GeneWise v2.4.1 (Birney et al. 2004) to build the initial gene predictions based on the best alignment information from the transcriptome or homologous proteins. We utilized Augustus v 3.3.3 software (Hoff and Stanke 2019) for model training and *de novo* gene prediction based on the initial gene predictions. Finally, we integrated the above results using EVidenceModeler v1.1.1 software (Haas et al. 2008) to generate the final annotation results. We assessed the completeness of this annotation using the BUSCO v5.4.3 (Manni et al. 2021) with the “tetrapoda_odb10” data set. We used the JCVI python package (Tang et al. 2008) to analyze and visualize collinearity between the four caecilian genomes.

To annotate the *Hox* genes, we first downloaded the protein sequences of *Hox* genes from the NCBI database (Sayers et al. 2023). We then applied the same method described above for homolog-based annotation to the genomes of 25 species (coelacanth, African lungfish, two-lined caecilian, Banna caecilian, Gaboon caecilian, tiny Cayenne caecilian, western clawed frog, axolotl, human, platypus, common wall lizard, mainland tiger snake, American alligator, spectacled caiman, Chinese alligator, saltwater crocodile, Darwin's rhea, emu, chicken, American flamingo, loggerhead sea turtle, common snapping turtle, Jardine River turtle, Yangtze giant softshell turtle, and yellow pond turtle; see supplementary fig S4 and table S15, Supplementary Material online). For conserved exon sequence in turtles and crocodiles, we used ssearch v36.3.8 h (Pearson and Lipman 1988) to map the CDS sequences of Banna caecilian *Hoxa14* gene to their genomes. Then, we extracted the exon sequences manually. MACSE v2.06 (Ranwez et al. 2018) was used to align the extracted exon sequences with parameters “-prog alignSequences.”

### Phylogenetic Reconstruction and Divergence Time Estimation

For the 24 species including human, mouse, platypus, American alligator, chicken, yellow pond turtle, sand microteiid, sand lizard, common wall lizard, glass lizard, central bearded dragon, python, Chinese habu, mainland tiger snake, Eastern brown snake, western terrestrial garter snake, corn snake, common toad, western clawed frog, axolotl, two-lined caecilian, Banna caecilian, Gaboon caecilian, and tiny Cayenne caecilian (supplementary table S15, Supplementary Material online), we first identified 2,755 single-copy orthologous genes using OrthoFinder v2.5.4 (Emms and Kelly 2019). The protein sequences of homologous genes were aligned using MAFFT v7.471 (Katoh and Standley 2013) with default parameters. For the species tree, we used IQ-TREE v1.6.12 (Nguyen et al. 2015) to construct a tree for each set of orthologous proteins with parameters “-m MFP -alrt 1000,” and the results were combined and analyzed using ASTRAL v5.7.1 software (Zhang et al. 2018) to reconstruct the species tree. We then used PAL2NAL v14 (Suyama et al. 2006) to convert protein alignments to CDS sequence alignments and extracted 4-fold degenerate sites (4D sites) for subsequently analysis. MCMCTree of the PAML v4.9j software suite (Yang 2007) was used for estimating divergence times, incorporating 3 records form TimeTree (Kumar et al. 2022) for fossil calibration (human–mouse: 87 Mya, human–chicken: 319 Mya, and human–common toad: 352 Mya). We then used the R8S v1.81 software (Sanderson 2003) to estimate mutation rates for each lineage, using the same data and calibration. Effective population sizes for the four caecilian species were estimated using the PSMC software (Li and Durbin 2011), with a generation time of 5 years (Wang et al. 2015). Heterozygosity analysis was performed using BCFtools v 1.10.2 (Danecek et al. 2021).

### Estimation of Increment of Intron Sizes

We collected gene sets from 17 species, including axolotl, bichir, chicken, coelacanth, common wall lizard, human, mouse, African lungfish, yellow pony turtle, python, platypus, spotted gar, thorny skate, two-lined caecilian, elephant shark, western clawed frog, and Banna caecilian (supplementary table S15, Supplementary Material online). Using mouse as a reference, we generated 1:1 orthologous gene set using the reciprocal best hit (RBH) method. The median lengths of gene introns and exons were calculated based on data from 14 vertebrate species, excluding axolotl, African lungfish, and Banna caecilian. Gene pairs were filtered out if the length of their exons differed by more than 40% from the median length, or if there were no introns in more than 6 species. For African lungfish, axolotl, and Banna caecilian, intron size expansion length was calculated by subtracting the median intron length. And the intron size expansion rate was calculated by dividing the median intron length. Genes with intron length less than twice the median length in African lungfish, Banna caecilian, and axolotl were identified as genes with limited intron length expansion and then subjected to GO enrichment analysis using mouse GO annotations from ENSEMBL BioMart data set (Kinsella et al. 2011) and hypergeometric distribution statistics in R, with a significance threshold of *P* value by false discovery rate (FDR) < 0.01.

To assess whether selective pressure influences intron length, we tested the hypothesis that genes with limited intron length in these 3 large genomes (African lungfish, Banna caecilian, and axolotl) are not randomly distributed across the genome. If selective pressure is indeed at work, we would expect to see a nonrandom pattern in the distribution of these genes, with more overlap between species than would occur by chance alone. To test this hypothesis, we employed Monte Carlo simulations, conducting 100,000 random trials. The simulation process was as follows: for each trial, we randomly selected genes to represent those with limited intron length, matching the observed numbers in each species (127 for African lungfish, 323 for Banna caecilian, and 327 for axolotl). We then calculated the number of genes that overlapped among these 3 species in each simulation. The distribution of overlap counts from these simulations was compared to the observed number of overlapping genes (49) in our data. This comparison allowed us to calculate a *Z*-score, quantifying the likelihood of observing such an overlap by chance and thereby assessing the strength of selective pressure on intron length across these species.

### Reconstruction of Ancestral Karyotypes

To reconstruct the ancestral karyotype of tetrapods, we utilized chromosome information from 8 species, including chicken, axolotl, western clawed frog, two-lined caecilian, Banna caecilian, Gaboon caecilian, tiny Cayenne caecilian, and the outgroup African lungfish. The reconstruction of ancestral karyotypes was conducted following the methods previously employed by Bi et al. (2021). We first used the RBH method to identify orthologous genes among these species. Then, using chicken chromosome information as a reference, we performed a χ^2^ test on the number of orthologous genes on different species' chromosomes, retaining only significantly conserved chromosomes and requiring a minimum of 20 orthologous genes. For the orthologous genes that meet the criteria, we used AGORA (Muffato et al. 2023) to determine syntenic blocks and used the results as input for ANGES (Jones et al. 2012) to reconstruct ancestral chromosomes. The final results were manually inspected.

### Identification of Limb-Related Gene Loss

To determine the loss of limb-related genes, we aligned the protein sequences of 21 species mentioned above (axolotl, sand microteiid, and glass lizard were excluded) with the mouse protein sequences as a reference. RBH method was used to identify the gene families to which the proteins belonged using mouse protein as reference. The limb-related genes were obtained from the gene database collected in the previous study (Roscito et al. 2022), resulting 732 genes in both human and mouse genome (supplementary table S8, Supplementary Material online). To ensure that gene loss was more likely to be associated with limb development, we selected only when those genes present in all tetrapod species with limbs for further analysis, resulting in 382 genes. We then carefully examined these 382 genes, requiring complete loss in all 6 snake species or all 4 caecilian species. Only the *Tulp3* gene, lost in snakes, and the *Grem1* gene, lost in caecilians, met the criteria.

To confirm the loss of limb-related genes, we conducted a manual homology annotation for each gene using the following steps: (i) protein sequence mapping: we used the tblastn program (with an *e*-value threshold of 1*e*−5) to map query protein sequences to the target genome (Camacho et al. 2009); (ii) region filtering and annotation: we filtered out regions that overlapped with known genes (such as other homologous proteins). We then annotated the remaining regions using GeneWise v2.4.1 software (Birney et al. 2004); (iii) phylogenetic analysis: we combined all annotated proteins with known proteins and reconstructed a phylogenetic tree. This step ensured that the gene loss occurred specifically in the query protein clade (He et al. 2019); and (iv) conservation analysis: to further verify the gene loss, we used VISTA to analyze and visualize the conservation of regions near the genes of interest (Frazer et al. 2004). These steps allowed us to systematically identify and confirm gene losses in our target species.

### Site-Specific Amino Acid Mutations

The same data set was employed for the analysis of shared changes in limb-related genes. Specifically, we required at least 7 conserved amino acids (AAs) within a region of 5 AAs upstream and downstream of sites that show specific changes in both snakes and caecilians, while being conserved across all limbed species (Wu et al. 2021, 2023).

### The Identification of CNEs and dCNEs

To identify tetrapod-conserved CNEs, we selected 43 tetrapod species for subsequent analysis. A detailed, step-by-step analysis is presented in supplementary fig. S6, Supplementary Material online. The data set includes 12 limbless species, 5 limbed amphibians, and 26 limbed amniotes (supplementary fig. S7a and table S15, Supplementary Material online). LAST v1282 software was used to perform pairwise whole-genome alignment with the human genome (GRCh38) as reference (parameters: lastdb -uMAM8; lastal -pHOXD70). Then, last-split program was used to get RBHs of each alignment. We obtained the phylogenetic relationship of these 43 species from TimeTree (Kumar et al. 2022) and manually checked the relationship among reptiles based on the study of Pyron et al. 2013. The final 43-way whole-genome alignment used Multiz v11.2 software with default parameters with the phylogenetic tree. The CNEs were identified using PHAST v1.5 package (Hubisz et al. 2011). In detail, we first calculated the evolutionary rate of 4D sites as nonconserved mod using msa_view program (parameters: –4d –features reference.gff) and phyloFit program (parameters: –EM –precision MED –subst-mod REV). Then, phastcons program (parameters: –target-coverage 0.3 –expected-length 45 –rho 0.3) was performed based on the nonconserved mod to get the conserved regions requiring the length more than 30 bp. Finally, 21,378 CNEs were identified after subtracting the coding region based on human annotation file and required that at least 29 limbed species existing. Genes that are located in the vicinity of CNEs within a 1 Mb range of their transcription start site were classified as related genes.

To identify dCNEs, we first reconstructed ancestral sequences. Following the method outlined in forward genomics (Prudent et al. 2016), we used PRANK v170703 software (parameters: -keep, -showtree, -showanc, -seed = 10; Löytynoja 2014) to reconstruct ancestral sequences at all nodes. Briefly, an “A” was added before the first base of each sequence (including those from species with complete sequence loss) to ensure all sequences were represented. After generating the ancestral sequences with PRANK, the added “A” was removed. This approach enabled us to reconstruct ancestral sequences at all nodes, even when sequences from some species were completely lost. We then employed a sliding window method (https://github.com/jackie-duang/SlideVar) to detect dCNEs based on 2 criteria: (i) a 20 bp window with 1 bp steps must have ≥90% (18 bp) sequence conservation in at least 29 limbed tetrapods compared to the reconstructed tetrapod ancestor sequence, while showing ≤70% (14 bp) sequence identity in limbless species, or (ii) a 50 bp window with 1 bp steps must have ≥60% (30 bp) sequence conservation in at least 29 limbed tetrapods but be absent in limbless species. A CNE was classified as degenerated if it met either criterion, allowing us to identify regions with significant evolutionary changes in limbless species while maintaining conservation in limbed tetrapods. To account for potential genome assembly quality issues, we required degeneration to occur in at least 3 snake species or 2 caecilian species to be considered a lineage-specific change. Additionally, we utilized forward genomics (parameters: –method = branch; Hiller et al. 2012) to analyze convergent dCNEs between any 2 limbless lineages.

To investigate the relationship between dCNE-associated genes and limb-related genes, we employed Fisher's exact test. For each combination of limbless lineages, the entire set of CNEs was used as the background. The rate of convergent dCNEs was calculated by dividing the number of convergent dCNEs in each limbless species by the time elapsed since limb loss in species.

The identity of convergent dCNEs in limbless species was calculated by comparing them to the reconstructed ancestor sequence of each limbless lineage. Additionally, we simulated sequence mutations based on neutral mutation rates at the same time scale using IQ-TREE v2.2.2.6 software (Nguyen et al. 2015) (parameters: –alisim -m GTR). The branch length of the input tree is calculated using the substation rates from the R8S software (Sanderson 2003) and then multiplied by the estimated divergence time. Ten simulations were performed for each CNE.

### Functional Annotation and Enrichment Analysis of Convergent dCNEs

We downloaded H3K27ac ChIP-seq data from ENCODE database (Luo et al. 2020) for limb tissues at different developmental stages in mice, the signal of which are commonly considered as potential enhancer sequences (supplementary table S10, Supplementary Material online). The H3K27ac ChIP-seq data from embryonic facial prominence, forebrain, heart, hindbrain, intestine, kidney, liver, lung, midbrain, neural tube, and stomach tissues in the ENCODE database were downloaded for pleiotropic analysis. For each CNE, we convert human positions to mouse positions using liftover (Kent et al. 2002) and consider it a potential enhancer region if there is an overlap with a database region where the -log_10_ (signal *P* value) is greater than 2. Based on this, we identified the enhancer activity of each CNE in different tissues.

We initially employed Fisher's exact test to determine whether the number of CNEs with limb enhancer signals among the convergent dCNEs was statistically significant. To investigate whether convergent dCNEs between snakes and caecilians are significantly associated with limb enhancers and at which embryonic development stages this association occurs, we utilized the LEG database (Monti et al. 2017) and ENCODE database. LEG database is based on computational methods for predicting limb enhancers. We then employed the LOLA package (Sheffield and Bock 2016) in R to perform genomic region enrichment analysis. In this analysis, the foreground consisted of convergent dCNEs shared by snakes and caecilians, while the background comprised all identified CNEs. We applied the Benjamini−Hochberg method for FDR correction to obtain the final results.

The Hi-C plot of CNE03754 and CNE17685 was generated using the Hi-C matrix data obtained from distal forelimbs and distal hindlimbs in a 12.5 d mouse embryo with GEO accession number: GSM2713716 (Rodríguez-Carballo et al. 2017). The HiCExplorer v3.7.2 software (Wolff et al. 2020) was utilized for this analysis.

### Identifying the Origin of CNEs and Selecting Experimented CNEs

To determine the origin of CNEs conserved in tetrapods, we aligned the corresponding human sequences to the genomes of African lungfish, coelacanth, bichir, spotted gar, bowfin, zebrafish, elephant shark, thorny skate, great white shark, hagfish, lamprey, and amphioxus (supplementary table S15, Supplementary Material online) using blastn (Camacho et al. 2009) sensitive parameters (-word_sizes 7 -evalue 0.001).

To identify elements for subsequent experimental validation, we applied the following filtering criteria: (i) the CNEs should be convergent dCNEs; (ii) the presence of enhancer signals during limb development in mice; (iii) the nearest coding genes adjacent to the CNEs must be limb-related genes; and (iv) given our interest in the contribution of younger CNEs to limb development, the origin of these CNEs should be more recent than the common ancestor of gnathostomes. We selected 3 of these CNEs, CNE03754, which emerged from the common ancestor of the common ancestor of lungfish and tetrapods; CNE17685, which emerged from the common ancestor of tetrapods; and CNE18083, which emerged from the common ancestor of osteichthyans, for further experiments.

### Generation of CNE Knockout Mice Using CRISPR/Cas9

The CNE knockout mouse strain was generated using an in vivo CRISPR/Cas9 editing method described in previous studies (Yang et al. 2014). The injection buffer mixture containing Cas9 mRNA at a concentration of 100 ng/μL and 2 single guide RNAs (sgRNAs) at concentrations of 25 ng/μL each was injected into the cytoplasm of single cell stage embryos derived from C57BL/6J strain mice. For the CNE03754 knockout experiment, the sgRNA sequences were 5′-TGTCGATATCTGCATCAGCTGGG-3′ and 5′-TGCCAGGAATGCTCCCGGAGTGG-3′. For the CNE17685 knockout experiment, the sgRNA sequences were 5′-GCCCCGGGGAAGGCAGTCGTCGG-3′ and 5′-GTCTCTTGACCTTTACTTCTGGG-3′. The last 3 nucleotides 5′-NGG-3′ are PAM sequences. Obtained F0 mice were validated by PCR and sequenced using the following primer pairs: for CNE03754, F1: 5′-GATGGAAAGCTGACTGATACAGCG-3′ and R1: 5′-GCCAAGATCCCTTGTCTTCTTGC-3′, and for CNE17685, F1: 5′-GGTGGAAATGCCTCAGTGGT-3′ and R1: 5′-GAGGAAGACAATATGCCTGGTGA-3′. F0 mice with expected deletions were chosen for further crossbreeding with WT mice to produce F1 mice. Homozygous mutant mice were finally generated through crossing.

### LacZ Transgenic Reporter Assay

LacZ transgenic reporter assay were performed as described in Zhuang et al. (2023). CNE18083 (chr13:15,696,633-15,696,840, mm10) was cloned into Hsp68-LacZ reporter vector. Embryos of transgenic mouse were generated by pronuclear injection, and F0 embryos were collected at E11.5 and stained for LacZ activity.

### Micro-CT Analysis

Mouse bones were fixed in 4% PFA for 48 h before scanning using micro-CT. Bone images were scanned by SkyScan1276 Micro-CT (Bruker Corporation, Billerica, MA) under conditions of voltage 46 kV, current 200 μA, and resolution 8.03 μm. Three-dimensional reconstructions of bones were generated using SkyScan CTVOX 1.5 software (Bruker Corporation, Billerica, MA). Trabecular and cortical parameters were evaluated using CTAn 1.20.8.0+ analysis software (Bruker Corporation, Billerica, MA).

### H&E Staining

The bones were fixed in 4% paraformaldehyde at room temperature for 48 h. They were demineralized by using 12% EDTA-2Na (Servicebio, Wuhan, China) for 4 weeks, dehydrated stepwise using ethanol, immersed in xylene, and embedded in paraffin. Paraffin was cut into 5 μm sections and then deparaffinized. The sections were stained with Mayer's H&E.

### Tol2 Transposon-Mediated Zebrafish Transgenesis Assay

The sequences of CNE03754 (chr9:48,866,503-48,866,845; mm10) and CNE17685 (chr12:34,660,214-34,660,410; mm10) were obtained through artificial synthesis. For each dCNE, the sequence was cloned into a plasmid vector containing a miniTol2 transposon donor, a mouse *cFos* basal promoter, and the coding sequence for green fluorescent protein (GFP). Subsequently, the plasmids harboring the target dCNEs were individually coinjected with transposase mRNA into WT zebrafish embryos at the 1- to 2-cell stage. Approximately 300 embryos were utilized for each CNE construct. GFP expression was monitored at 24 h intervals post-fertilization. A dCNE was considered to exhibit specific enhancer activity only if consistent GFP expression was observed in more than 20% of the injected embryos.

## Results and Discussions

### Large Genome Assembly of Banna Caecilian

**Fig. 1.**
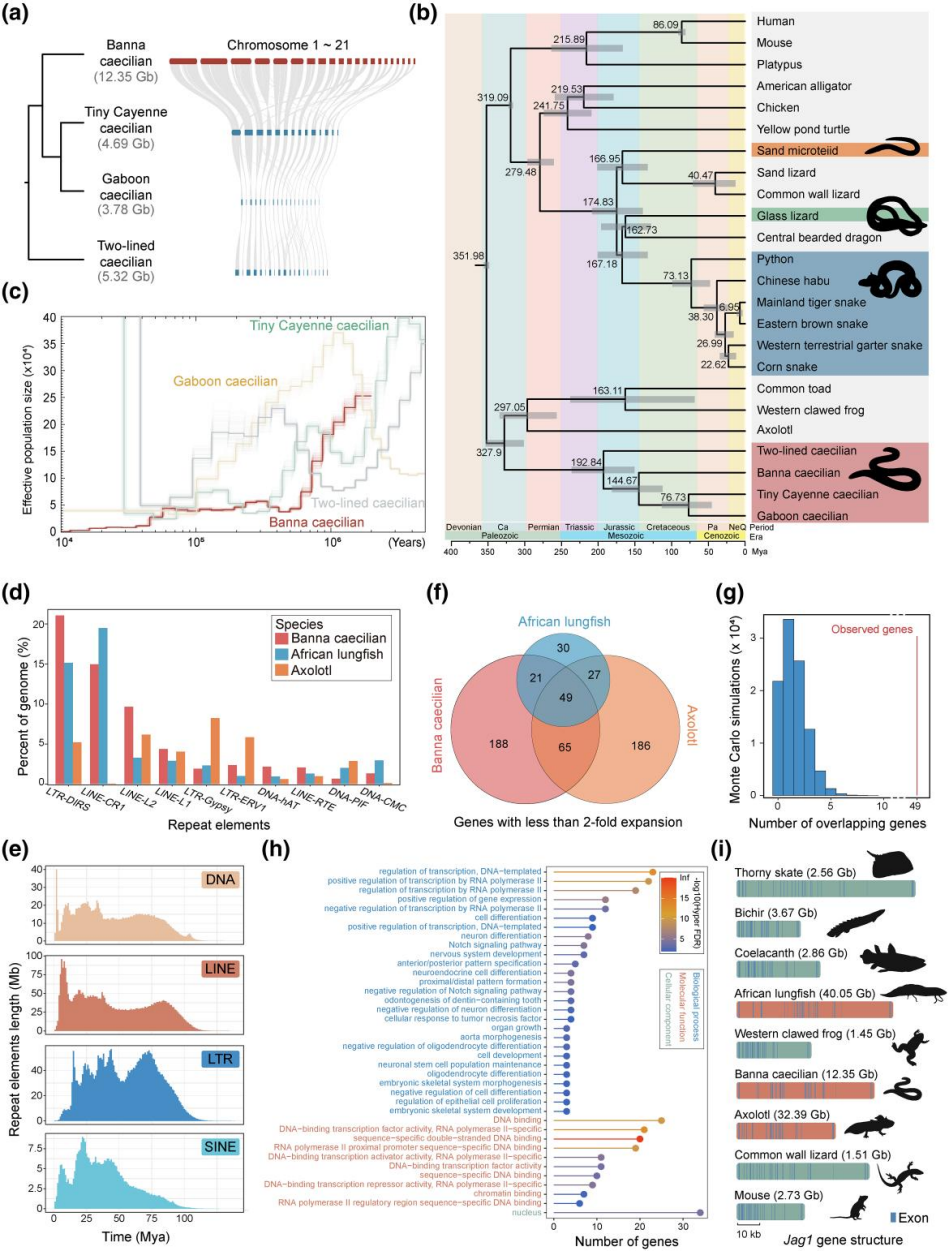
The phylogenetic relationships of caecilians and genome expansion in Banna caecilian. a) Conserved synteny relationships among 4 Gymnophiona genomes. Genome size of these species is various as shown after the species name. b) Reconstruction of phylogenetic relationships for 24 species and estimation of divergent times. c) Estimation of the recent population history of 4 caecilians. d) The major types of TE families in the genomes of different species are distinct. e) Estimation of the amplification time of TE families. f) Venn diagram showing the number of genes with limited intron length in African lungfish, axolotl, and Banna caecilian. g) Distribution of overlapped genes with limited intron length from 100,000 Monte Carlo simulations among African lungfish, axolotl, and Banna caecilian. The solid line marks the real number. h) GO enrichment results for 49 shared genes with limited intron length. i) Gene structure of *Jag1* in different species. In brackets are the genome sizes of the corresponding species.

The genome size of Banna caecilian was estimated to be 12.5 Gb through the analysis of *k*-mer frequency of short reads (supplementary fig. S1a, Supplementary Material online), close to the size of 12.2 Gb determined in a prior study using flow cytometry (Wang et al. 2021). We generated 478 Gb of long reads (∼38× coverage, N50 read length ∼14.67 kb) using Pacbio SMRT sequencing, 780 Gb (∼62×) of short reads, and 631 Gb (∼50×) of Hi-C reads (supplementary table S1, Supplementary Material online). The assembly was performed using NextDenovo (Hu et al. 2024) and All-HiC (Zhang et al. 2019). The final genome assembly comprises 21 chromosomes (Fig. 1a; supplementary fig. S1b, Supplementary Material online). The size of the final assembly is 12.35 Gb, with contig and scaffold N50 of 5.7 Mb and 1.04 Gb, respectively (supplementary table S2, Supplementary Material online), represents one of the largest published vertebrate genomes (Nowoshilow et al. 2018; Meyer et al. 2021; Schloissnig et al. 2021; K. Wang et al. 2021; Schartl et al. 2024). The assembled chromosomes, ranging in size from 144 Mb to 1.41 Gb, are consistent with the karyotype previously reported for this species (supplementary fig. S1c, Supplementary Material online) (Wen and Pang 1990).

The genome assembly was found to be highly accurate, with 99.9% of the short reads mapping successfully and 99% of the bases achieving more than 20-fold coverage depth from combined short and long reads (supplementary fig. S1d, Supplementary Material online). Furthermore, the 481 vertebrate ultraconserved elements (UCEs) identified in an earlier study (Bejerano et al. 2004) were used to assess genome assembly integrity. The results show that 436 UCEs (90.64%) are present in the Banna caecilian genome. In comparison, 409 (85.0%), 401 (83.4%), and 394 (81.9%) UCEs are present in tiny Cayenne caecilian (*Microcaecilia unicolor*) (Ovchinnikov et al. 2023), Gaboon caecilian (*Geotrypetes seraphini*) (Ovchinnikov et al. 2023), and two-lined caecilian (*Rhinatrema bivittatum*) (Rhie et al. 2021), respectively. Based on the homologous proteins of vertebrates and transcript data set, a total of 23,143 genes were annotated, including 5,029 (94.7%) complete conserved orthologs within tetrapods, based on BUSCO analysis (supplementary table S3, Supplementary Material online). The highly conserved synteny observed between the Banna caecilian genome and the genomes of the 3 other published caecilian species demonstrates that homologous chromosome segments are clearly identifiable across these four Gymnophiona species, despite the significant variation in genome size: Banna caecilian (12.35 Gb), tiny Cayenne caecilian (4.69 Gb), Gaboon caecilian (3.78 Gb), and two-lined caecilian (5.32 Gb), as illustrated in Fig. 1a.

The phylogenetic relationship between the Banna caecilian and other species were reconstructed utilizing protein coding genes from 24 species (Fig. 1b; supplementary fig. S1e, Supplementary Material online). The two-lined caecilian represents the basal lineage of the order Gymnophiona, while the Banna caecilian forms a sister clade with the Gaboon caecilian and tiny Cayenne caecilian. It was estimated that Gymnophiona diverged from the common ancestor of Urodela and Anura around 328 Mya aligns with recent studies on fossils from the Late Triassic epoch (Kligman et al. 2023). The divergence time between different caecilians suggests that the loss of limbs occurred no later than 192 Mya, earlier than in snakes (∼170 Mya; Garberoglio et al. 2019) and much earlier than in the sand microteiid (*Calyptommatus sinebrachiatus*) and glass lizard (*Dopasia gracilis*) (∼35 and 40 Mya; Roscito et al. 2022). In addition, the effective population size of Banna caecilian experienced the most significant decline since 50 thousand years ago compared to other caecilians (Fig. 1c). Despite being listed as “Least Concern” by IUCN, the genetic diversity of this species is the lowest of all sequenced caecilians (supplementary fig. S1f, Supplementary Material online). More attention needs to be paid to the conservation and understanding of Banna caecilian.

Like the African lungfish (*Protopterus annectens*; ∼40 Gb) and the axolotl (*A. mexicanum*; ∼32 Gb), the Banna caecilian has not undergone any additional whole-genome duplication events beyond the 2R-WGD shared by jawed vertebrates (supplementary fig. S2a, Supplementary Material online) (K. Wang et al. 2021; Marlétaz et al. 2024). Its large genome size is mainly due to transposable element (TE) amplification, which makes up 79% (9.78 Gb) of the genome (supplementary table S4, Supplementary Material online). However, the predominant TE classes vary among these large-genome species: LTR-DIRS (21%) in Banna caecilian, LINE-CR1 (19%) in African lungfish, and LTR-Gypsy (8%) in axolotl (Fig. 1d). We traced the evolutionary history of these TEs in the Banna caecilian and found that different types of TEs exhibit distinct amplification patterns over time. DNA, LINE, and SINE TEs showed amplification peaks within the last 20 million years, likely contributing to progressive genome expansion (Fig. 1e). Despite overall intron size increase (supplementary fig. S2b to e, Supplementary Material online) (Wang et al. 2021), 49 genes, 27 of which encode transcription factors, show limited intron expansion, with an amplification ratio less than twice the median intron length across 14 vertebrate species (Fig. 1f; supplementary table S5, Supplementary Material online). A Monte Carlo simulation confirmed that the limited intron expansion observed in these genes is statistically significant (*Z*-score = 39.17; *P* value = 0; Fig. 1g). Gene Ontology (GO) enrichment analyses showed that they were significantly associated with growth and development, encompassing cell differentiation, neuron differentiation, nervous system development, and embryonic skeletal system morphogenesis (adjusted *P* < 0.01) (Fig. 1h; supplementary table S6, Supplementary Material online). A notable example is the *Jag1* gene, essential for embryo development (Grochowski et al. 2016), with a long median intron length across 14 vertebrate species (41.6 kb) and numerous introns (∼25). Unlike similarly sized genes, *Jag1*'s introns show minimal variation in median length (Figs. 1i; supplementary S2f and g, Supplementary Material online). Similar conservation of intron size in developmental genes was observed in axolotl (Nowoshilow et al. 2018; Lertzman-Lepofsky et al. 2019). This suggests that intron length may influence transcription efficiency and organismal development (Swinburne et al. 2008).

**Fig. 2.**
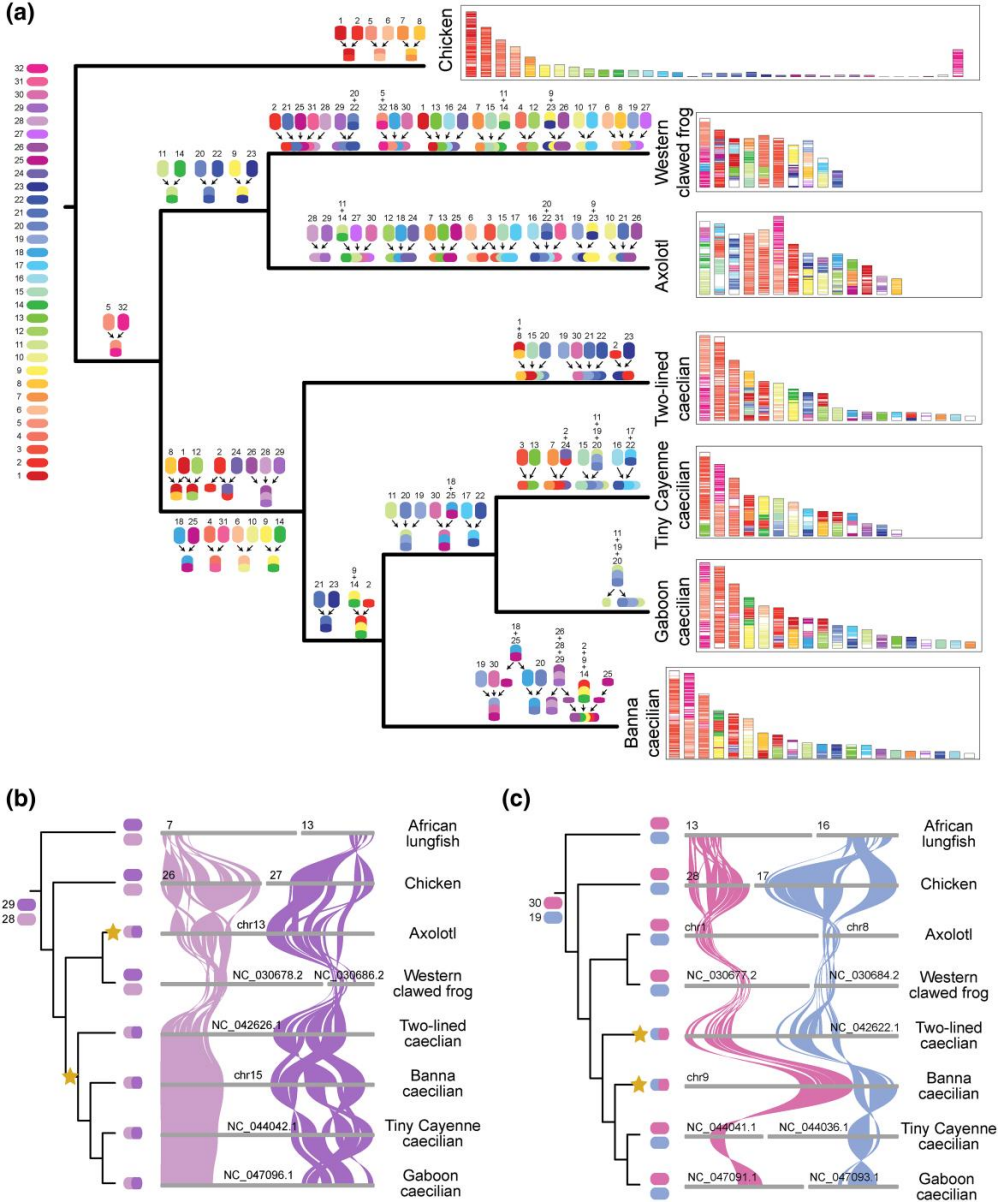
Reconstruction and evolution of proto-chromosomes in Gymnophiona. a) Reconstruction of the 32 proto-chromosomes of the last common ancestor of tetrapods. b and c) Demonstration of 2 independent fusion events occurring in different lineages. Asterisks indicate nodes where fusion events occurred.

Using high-quality chromosome information, we further explored the karyotype evolution of amphibians (Fig. 2a; supplementary table S7, Supplementary Material online). A total of 32 and 31 proto-chromosomes were reconstructed for the last common ancestor of tetrapods and amphibians, respectively. For the last common ancestor of Gymnophiona, 8 fusion events and 2 fission events were observed. Additionally, we observed independent fusion events of proto-chromosomes 28-29 in the axolotl and the last common ancestor of Gymnophiona, as they remained unfused in African lungfish, chicken, and western clawed frog (Fig. 2b). Another hypothesis of the fusion events is that fusion occurred in the last common ancestor of amphibians, followed by fission in western clawed frog. Given that there are 2 independent proto-chromosomes, this implies that the fission event must occur precisely between the 2 proto-chromosomes, rather than being an intrachromosomal fission, such as the fission between proto-chromosomes 1 or 2 in the last common ancestor of Gymnophiona. This places greater constraints on the fission event. Therefore, we are not inclined to this hypothesis. Similarly, fusion events involving proto-chromosomes 19-30 were observed in the two-lined caecilian and Banna caecilian, whereas no fusion occurred in the tiny Cayenne caecilian, Gaboon caecilian, and all other four species (Fig. 2c). The chromosome-level assembly of Banna caecilian provides an important genetic resource for studying the evolution of Gymnophiona.

### Absence of Limb-Related Genes in Limbless Tetrapods

**Fig. 3.**
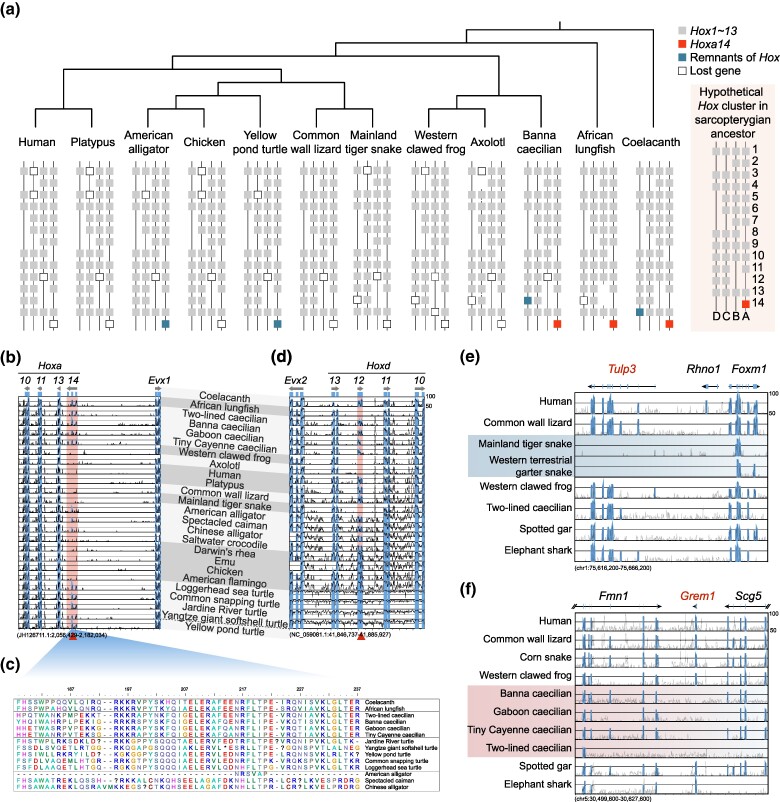
*Hox* gene clusters in caecilians and the absence of limb-related genes in snakes and caecilians. a) Schematic representation of the *Hox* gene clusters in sarcopterygians. b) The VISTA plot of the *Hoxa14* gene. *Hoxa14* sequences have conserved sequences in caecilians, crocodiles, and turtles. The genome of the coelacanth (LatCha1) was used as a reference. *Hoxa14* gene is shown in shadow. c) Results of the partial amino acid sequence alignment of *Hoxa14*. “?” represents frameshift mutation sites, and “*” represents premature stop sites. d) The VISTA plot of the *Hoxd12* gene. The genome of the yellow pond turtle (GCF_020497125.1) was used as a reference. *Hoxd12* gene is pseudogenized in caecilians, despite of remained conservation. *Hoxd12* gene is shown in shadow. e and f) The VISTA sequence conservation plot shows the loss of the *Tulp3* gene in snakes and *Grem1* gene in caecilians. The genome of the chicken (galGal5) was used as a reference.

To explore the genetic basis of limb loss, we first focused on *Hox* gene clusters, essential for regulating the body plan during embryonic development, including limb development. We identified the *Hoxa14* gene in all 4 caecilian genomes (Figs. 3a; supplementary figs. S3 and S4, Supplementary Material online), which was thought to be lost in the common ancestor of tetrapods (Pascual-Anaya et al. 2013). Additionally, we found conserved exon sequences in turtles and some crocodilians, despite that these sequences have undergone pseudogenization (Fig. 3b and c). Notably, the pseudogenization of *Hoxd12* gene in caecilians and the absence of *Hoxd12* gene in snakes, as well as in western clawed frog and axolotl, suggest that it is earlier lost in the common ancestor of amphibians (Fig. 3d). Thus, there is no evidence that alterations in the *Hox* gene clusters caused the loss of limbs.

We carefully examined 382 limb-related genes from the database collected in previous study (Roscito et al. 2022), investigating their presence or absence in caecilians and snakes (supplementary table S8, Supplementary Material online). While the majority of these genes were found to be present in both lineages, we identified several notable exceptions that highlight the complex genetic underpinnings of limb loss in different tetrapod groups. Our analysis revealed 2 notable findings: the absence of *Tulp3* in snake genomes and the absence of *Grem1* in caecilian genomes (Fig. 3e and f). TULP3 plays a crucial role in proper hedgehog signaling, interacting with the core SHH pathway to regulate anterior-posterior patterning during early limb bud formation (Cameron et al. 2009). It also influences primary cilia function, which are essential organelles for developmental signaling (Norman et al. 2009). *Tulp3* loss leads to embryonic lethality at E14.5 and altered anterior-posterior axis patterns in early limb bud development in C57BL/6J mice (Ikeda et al. 2001; Cameron et al. 2009). Furthermore, the gene *Grem1* was found to be absent in all 4 examined caecilian genomes (Fig. 3f). GREM1, on the other hand, acts as a key BMP antagonist in the limb bud. It forms a feedback loop with FGF signals from the AER to regulate proximodistal outgrowth. In the posterior limb bud, GREM1 interacts with SHH from the ZPA to maintain the SHH-FGF feedback loop, critical for proper limb development (Khokha et al. 2003; Michos et al. 2004). *Grem1* knockout mice show varying degrees of limb defects, from embryonic lethality to severely shortened limbs, depending on their genetic background (Canalis et al. 2012). Interestingly, while *Grem1* is absent in all 4 examined caecilian genomes, it is present in snakes and 2 limbless lizard species (glass lizard and sand microteiid) (Fig. 3f). This differential gene loss pattern underscores the complexity and diversity of genetic changes underlying limb loss in different tetrapod lineages. Additionally, we found that *Twist2*, previously known to be absent in snakes (Roscito et al. 2022), is also missing in all amphibian species. Its loss may represent a broader pattern of gene loss in certain tetrapod lineages. Moreover, we also identified several genes, including *Tbx5* and *Bmpr1a*, exhibiting mutations at identical sites across multiple lineages (supplementary fig. S5 and table S9, Supplementary Material online). While these mutations occur at the same positions, further investigation is needed to determine if they represent true convergent evolution.

In conclusion, the retention of most limb-related genes in limbless species likely reflects their pleiotropic roles in embryonic development beyond limb formation. The specific losses of *Tulp3* in snakes and *Grem1* in caecilians, along with the broader absence of *Twist2* in amphibians and snakes, highlight the diverse genetic pathways that can be affected by evolutionary pressures in different tetrapod lineages, some of which may contribute to limb loss.

### Convergent Degenerated Conserved Noncoding Elements in Limbless Tetrapods

**Fig. 4.**
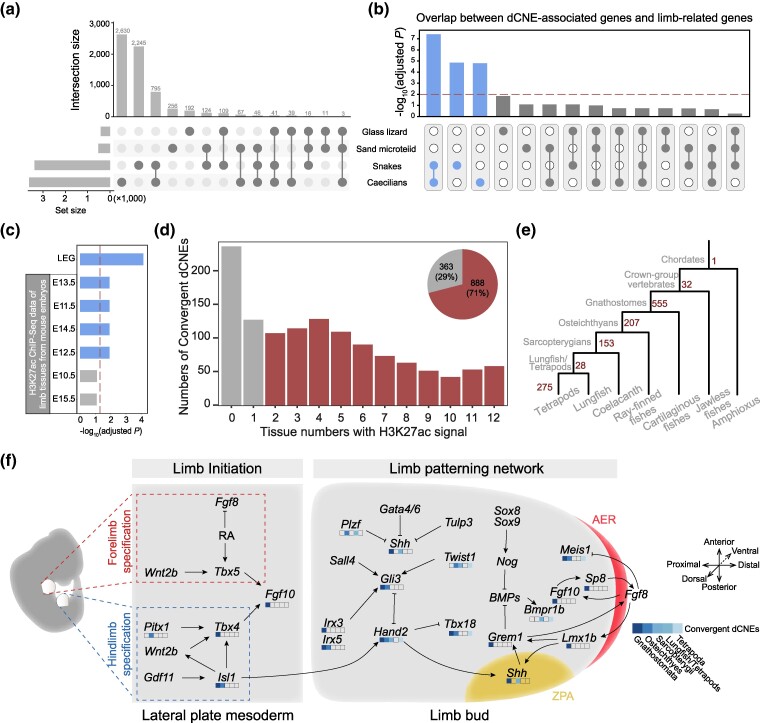
Convergent dCNEs are candidate limb regulatory elements and originating at different evolutionary time points. a) UpSet plot of 6,574 dCNEs across 4 limbless lineages. b) The significance of overlap between dCNE-associated genes and limb-related genes across different combinations of limbless lineages. The dashed line indicates an adjusted *P* value of 0.01. c) Enrichment analysis of 882 convergent dCNEs between snakes and caecilians showing significant overlap with limb-related databases. The dashed line indicates an adjusted *P* value of 0.05. d) Convergent dCNEs exhibit H3K27ac signals across multiple tissues in developing mouse embryos, with most of these dCNEs displaying pleiotropic effects (n = 888, 71%). The pie chart in the upper right corner illustrates the proportions of convergent dCNEs with different pleiotropic effects. e) The numbers of convergent dCNEs at different origins. f) Some convergent dCNEs with different origins are neighbor to genes involved in limb development pathway. ZPA, zone of polarizing activity; AER, apical ectodermal ridge.

To further explore genomic changes in limb-related regulatory elements in limbless species, we constructed a 43-way whole-genome alignment using 12 limbless tetrapods and 31 tetrapods with limbs (supplementary figs. S6 and S7a, Supplementary Material online). Based on the alignment, we identified 21,378 conserved noncoding element (CNEs) in tetrapods with limbs, present in at least 29 of the 31 species, with lengths ranging from 30 to 3,371 bp (supplementary fig. S7b, Supplementary Material online). We then identified degenerated CNEs (dCNEs) as those with no more than 70% sequence identity in limbless species but at least 90% identity in limbed tetrapods within a 20 bp window. Alternatively, dCNEs could also be completely lost in limbless species within a 50 bp window, while retaining at least 60% identity in limbed tetrapods. A total of 6,574 dCNEs were identified across 4 limbless lineages, including 4 caecilians, 6 snakes, and 2 limbless lizards (Fig. 4a). Most dCNEs (5,323) are lineage-specific, particularly in caecilians and snakes, which account for roughly three-quarters of these dCNEs.

We focused on convergent dCNEs within at least 2 lineages and identified a total of 1,251 convergent dCNEs. The majority of these dCNEs (1,145) were convergent between 2 lineages, while the remainder (106) were convergent among 3 lineages. Notably, no dCNE was found to be convergent among all 4 lineages. Similar to lineage-specific dCNEs, caecilians and snakes have the majority of convergent dCNEs (*n* = 882, 70.5%), while limbless lizards account for only a small portion (*n* = 30, 2.4%). We examined whether there was a significant overlap between dCNE-associated genes and limb-related genes. The results showed that the convergent dCNEs between snakes and caecilians exhibited the strongest significance (Fig. 4b). Furthermore, it indicates that limb-related regulatory elements in snakes and caecilians have undergone convergent degeneration following limb loss in these lineages.

To further investigate the role of these convergent dCNEs in limb development, we utilized ChIP-seq data of H3K27ac in mouse limb from the ENCODE database (supplementary table S10, Supplementary Material online) (Luo et al. 2020), which represents potential enhancer signals. We identified 546 convergent dCNEs with enhancer signals associated with limb development, which are significantly enriched (*P* = 0.0001) compared to all CNEs. Most of them (*n* = 387) are convergent between caecilians and snakes (supplementary fig. S7c, Supplementary Material online). Further analysis shows that convergent dCNEs in caecilians and snakes are significantly enriched in Limb-Enhancer Genie (LEG), a computation-based limb enhancer database (Monti et al. 2017). This enrichment was also observed during mouse limb development at E13.5, E11.5, E14.5, and E12.5 stages (Fig. 4c). These results suggest that convergent dCNEs may act as limb-related regulatory elements, as most have not been thoroughly investigated.

The high number of convergent dCNEs we found suggests that most are not driving limb loss. Instead, they likely reflect relaxed selection on limb-related regions after limblessness evolved independently in these tetrapod lineages. The abundance of convergent dCNEs in caecilians and snakes compared to the relatively few convergent dCNEs in limbless lizards may be influenced by the time elapsed since limb loss. We calculated the rates of convergent dCNEs per million years for different limbless species, finding the following results: caecilians (3.07 to 4.77), snakes (3.9 to 6.36), glass lizard (5.475), and sand microteiid (7.63) (supplementary table S11, Supplementary Material online). A median rate of 5.635 suggests that more convergent dCNEs accumulate over longer evolutionary timescales. This finding is also consistent with the hypothesis of relaxed natural selection (Roscito et al. 2022). To ensure that these inferences are not influenced by potential biases in our pipeline, we used forward genomics (Hiller et al. 2012), another software for identifying convergent dCNEs, to explore convergent dCNEs between different lineages. Consistently, snakes and caecilians exhibit the highest number of convergent dCNEs (supplementary table S12, Supplementary Material online). In addition, to assess whether evolutionary time is the sole reason for the observed changes in CNEs, we conducted a comparative analysis in limbless species. We examined non-dCNEs, convergent dCNEs, and simulated sequences evolving under neutral drift over equivalent time periods. Histogram distributions revealed distinct patterns for each group, and *t*-tests showed statistically significant differences among all 3 groups (supplementary fig. S8, Supplementary Material online). These findings indicate that the observed evolutionary patterns cannot be solely attributed to neutral drift. The differential evolutionary rates among non-dCNEs, convergent dCNEs, and neutrally evolving sequences suggest the presence of distinct selective pressures, potentially reflecting the pleiotropic nature of CNEs as candidate regulatory elements. This hypothesis is supported by ENCODE data (Luo et al. 2020), which shows that most (*n* = 888, 71%) of the convergent dCNEs exhibit strong enhancer activity in multiple tissues during mouse development (Fig. 4d).

Although most convergent dCNEs appear to result from relaxed selection, their conservation in limbed species and convergent degradation in independent limbless lineages strongly indicate their involvement in limb development. Moreover, given their early origin in the common ancestor of tetrapods, these convergent dCNEs likely represent a crucial genetic basis for limb formation in this ancestor.

### Limb Development-Related CNEs Originating at Different Evolutionary Time Points

To investigate the roles of convergent dCNEs in limb evolution, we traced their evolutionary history (Fig. 4e) and potential interaction genes (Fig. 4f; supplementary table S13, Supplementary Material online). Most of these dCNEs (*n* = 555, 44.36%) emerged in the gnathostome ancestor, around the time paired fins first appeared and later evolved into tetrapod limbs (Coates 2003). Two of these convergent dCNEs have been experimentally linked to limb development. The ZRS enhancer, a key regulator of *Shh* during limb formation (Lettice et al. 2002), is absent in caecilians and degraded in snakes (supplementary fig. S9a, Supplementary Material online). Similarly, LARM1, a crucial limb-specific enhancer of *Lmx1b* essential for limb dorsalization in mice (Haro et al. 2021), is missing in all 6 snakes and 2 caecilians examined (supplementary fig. S9b, Supplementary Material online). These findings suggest that these ancient convergent dCNEs are critical for the development and patterning of paired appendages, including limbs.

**Fig. 5.**
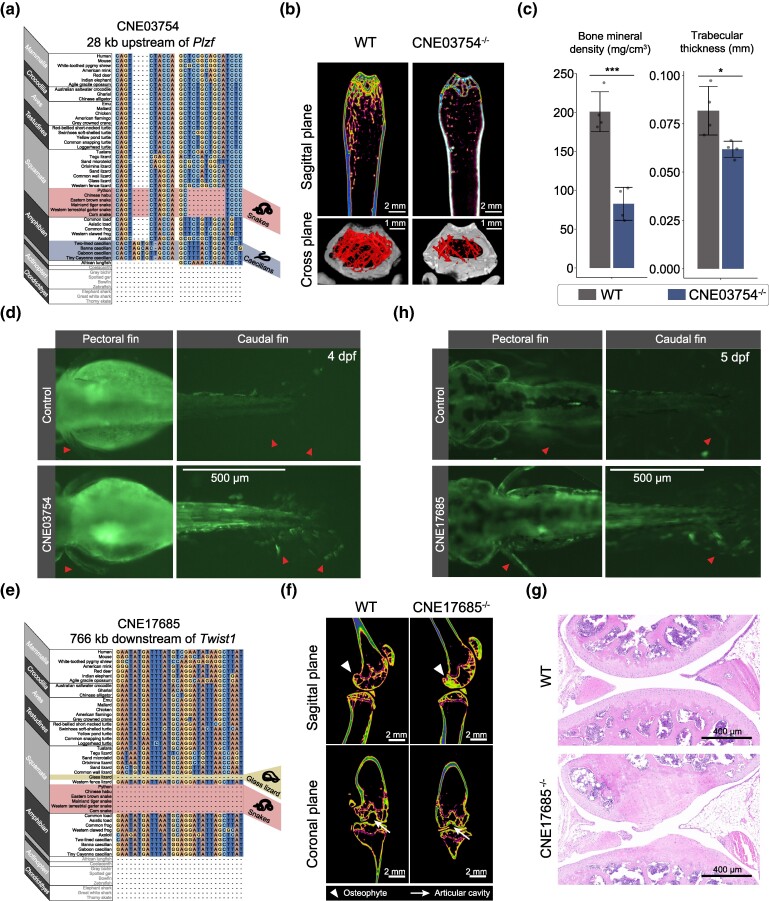
Functional experiments of convergent dCNEs at different origins. a) Partial sequence alignment of CNE03754 (chr11:114,031,289-11,4031,631, hg38), located 28 kb upstream of *Plzf*, which originates from the common ancestor of tetrapods and lungfish, degenerated in both snakes and caecilians. b) Micro-CT images of the femur in WT and CNE03754^−/−^ mice (*n* = 4). Images captured in both the sagittal plane and the cross plane show a significant reduction in bone density in CNE03754^−/−^ mice compared to WT mice. c) Statistics of bone density and trabecular thickness in WT and CNE03754^−/−^ mice. The data are shown as the mean ± SD (standard deviation). *P < 0.05; ***P < 0.001. d) Reporter assay in zebrafish embryos shows that the CNE03754 has enhancer activity in pectoral fins (left panel) and caudal fins (right panel) at 4 dpf. e) Partial sequence alignment of CNE17685 (chr7:18,350,679-18,350,876, hg38), located 766 kb downstream of *Twist1*, which originates from the common ancestor of tetrapods, lost in both snakes and glass lizard. f) Micro-CT images show significant cartilage defects (the triangle in the upper panel) and reduced foramina (the arrow in the lower panel) in the CNE17685^−/−^ mice (*n* = 3). g) H&E staining of WT and CNE17685^−/−^ mice (*n* = 3). WT mice have smooth articular surfaces and normal synovial interstitial cell structure. However, the articular surfaces of CNE17685^−/−^ mice are uneven and show marked hyperplasia of the synovial lining and matrix cells. h) Reporter assay in zebrafish embryos shows that the CNE17685 has enhancer activity in pectoral fins (left panel) and caudal fins (right panel) at 5 dpf.

Additional convergent dCNEs arose later: 207 in osteichthyans, 153 in sarcopterygians, 28 in the lungfish-tetrapod ancestor, and 275 in tetrapods (Fig. 4e). These newer CNEs may influence derived limb features. To explore this hypothesis, we selected 2 convergent dCNEs for functional testing in limb development. The first CNE (CNE03754, located in UCE7559) (Alexander et al. 2017) emerged in the common ancestor of lungfish and tetrapods but has since degenerated in both snakes and caecilians (Fig. 5a). To investigate the function of CNE03754 (chr9:48,866,503-48,866,845, mm10), we generated knockout mice using CRISPR/Cas9 (CNE03754^−/−^, chr9:48,866,340-48,866,894 deleted with 2 bp [AT] inserted; supplementary fig. S10a to c, Supplementary Material online). CNE03754^−/−^ mice exhibited significantly reduced bone density compared to wild-type (WT) controls (Figs. 5b and c; supplementary fig. S10d and e and table S14, Supplementary Material online). Other than reduced bone density, no significant phenotypic abnormalities were observed in the adult mice, suggesting that CNE03754's function is not primarily related to limb morphology, unlike ZRS or LARM1. To further validate enhancer effect of CNE03754, we conducted transgenesis reporter assays in zebrafish. Interestingly, the expression pattern of GFP was detected in the pectoral and caudal fins at 4 d post-fertilization (dpf) (Fig. 5d). Since CNE03754 is not naturally present in zebrafish, these findings confirm its role as a limb-related enhancer. They also suggest a level of conservation in regulatory elements involved in vertebrate limb development. CNE03754 is situated at 28 kb upstream of the *Plzf* gene, a potential target. Hi-C data from E12.5 mouse limbs demonstrate that CNE03754 and *Plzf* colocalize within a topologically associated domain (TAD) (supplementary fig. S10a, Supplementary Material online). The *Plzf* gene, also known as *Zbtb16*, is a member of the POZ/zinc finger family of transcription factors and plays a crucial role in limb development by regulating the proliferation and differentiation of limb bud mesenchymal cells (Suliman et al. 2012). *Plzf* expression is decreased in the mesenchymal stem cells of osteoporotic mice, and upregulating the *Plzf* gene promotes osteogenic differentiation (Yu et al. 2023). The osteoporotic phenotype observed in CNE03675^−/−^ mice suggests that CNE03754 may regulate bone mass by modulating *Plzf* gene expression.

The other CNE (CNE17685; chr12:34,660,214-34,660,410, mm10) originates from the common ancestor of tetrapods and is absent in snakes and glass lizards (Fig. 5e). Mice with CRISPR/Cas9-mediated knockout of this CNE (CNE17685^−/−^; chr12:34,660,209-34,660,389 deleted; supplementary fig. S11a to c, Supplementary Material online) tended to develop an arthritic phenotype. Micro-computerized tomography (micro-CT) scans revealed that knockout mice developed subchondral bone sclerosis and osteophytes at the femoral condyle, while normal mice showed no osteophytes (Fig. 5f, top). Additionally, micro-CT scans in the coronal orientation showed that CNE17685^−/−^ mice had decreased joint spacing in the articular cavity (Fig. 5f, bottom). Validation of micro-CT scans was carried out using hematoxylin and eosin (H&E) staining and safranin fast green staining (Figs. 5g; supplementary S11d, Supplementary Material online). CNE17685^−/−^ mice displayed changes in articular morphology, including thickening of the articular cartilage, altered chondrocyte structure, and compromised surface integrity. We also performed transgenesis reporter assays for this CNE in zebrafish. The expression pattern driven by CNE17685 was observed in the pectoral and caudal fins at 5 dpf (Fig. 5h), confirming its role as an enhancer in vertebrate appendage development. CNE17685 is located downstream of the gene *Twist1* and the intron region of *Hdac9* gene and located within the same TAD with *Twist1* in E12.5 of mouse limb (supplementary fig. S11a, Supplementary Material online). Many enhancers in the intronic region of *Hdac9* have been shown to regulate *Twist1* expression in previous studies (Hirsch et al. 2018). The gene *Twist1* is critical for limb and cartilage development, with knockout leading to embryonic lethality at E11.5. Altered *Twist1* activity can result in significant defects such as polydactyly, limb abnormalities, and various skeletal malformations in mice (Chen and Behringer 1995; Krawchuk et al. 2010). Another experiment on chondrocyte differentiation using human bone marrow mesenchymal stem cells demonstrated that *Twist1* expression is dynamically regulated, with proper chondrogenic differentiation requiring initial upregulation followed by downregulation of *Twist1* expression (Cleary et al. 2017). The abnormal cartilage differentiation phenotype observed in CNE17685^−/−^ mice aligns with the findings on *Twist1* function on chondrogenic differentiation. CNE17685 may influence the expression pattern of *Twist1* during chondrogenic development.

In addition to the 2 convergent dCNEs mentioned above, several others are notable. For example, 1 convergent dCNE (CNE07845; supplementary fig. S12a, Supplementary Material online) is located 82 kb downstream of *Tbx4*, which is the core gene responsible for the initial development of the hindlimbs (Rodriguez-Esteban et al. 1999). Additionally, the regulation of *Gli3* expression plays a crucial role in both fin and limb development. In tetrapods like mice and chickens, *Gli3* expression is restricted to the anterior region of the developing limb bud, while in fish such as the small-spotted catshark (*Scyliorhinus canicula*), it remains broadly distributed across the fin bud (Onimaru et al. 2015). We identified 2 convergent dCNEs, CNE18083 and CNE18098 (supplementary fig. S12b, Supplementary Material online), located within the intronic region of *Gli3*, which may influence its expression patterns. Another dCNE (CNE14635, supplementary fig. S12c, Supplementary Material online) is located 71 kb upstream of *Hand2*, which is important for the specification of the pentadactyl autopod (Galli et al. 2010). CNE18083 was selected for enhancer activity testing as it is absent in 2 caecilians and 5 snakes. LacZ transgenic reporter assays revealed enhancer activity for CNE18083 in the developing limb buds of E12.5 mouse embryos, with prominent staining visible in both the forelimb and hindlimb regions (supplementary fig. S12d, Supplementary Material online). Collectively, these findings demonstrate that convergent dCNEs from diverse evolutionary origins serve as critical regulatory elements in both limb and fin development, offering new insights into the genetic mechanisms underlying limb evolution and loss across vertebrate lineages.

## Conclusion

The phenomenon of convergent limb loss in tetrapods provides valuable insight into the genetic and developmental processes shaping phenotype (Leal and Cohn 2016; Infante et al. 2018; Wu et al. 2021). Our comprehensive analysis of the Banna caecilian genome, coupled with comparative genomics across limbless tetrapods, has revealed several key insights into the genetic mechanisms underlying limb loss and development. The exceptionally large genome of the Banna caecilian (12 Gb) exhibits an intriguing pattern of gene length variation. While most genes tend to increase in length with genome expansion, a subset of developmental genes, particularly transcription factors, shows relatively limited length expansion. This pattern suggests a balance between genome expansion and the need to preserve the functional integrity of key developmental genes, highlighting the selective pressures that limit gene length changes in transcription factors.

Our investigation into the genetic impact of limb loss focused on both limb-related genes and regulatory elements. In limbless species, the loss of key limb development genes is infrequent but does occur in some cases. For example, the loss of essential limb development genes like *Grem1* in caecilians and *Tulp3* in snakes has been observed, even though these genes play crucial roles in other developmental processes. This suggests that functional redundancy or compensatory mechanisms may have evolved in these lineages to offset the loss of these genes. In contrast, we observed more widespread degeneration of regulatory elements, particularly convergent dCNEs linked to limb development.

Most of these convergent dCNEs originated in the common ancestor of jawed vertebrates, around the time paired fins or limbs first evolved. This observation highlights the deep homology between limbs and fins, emphasizing the crucial role these regulatory elements play in appendage evolution (Adachi et al. 2016). Our functional experiments further confirm that relatively newer convergent dCNEs are associated with limb development and may have contributed to the derived characteristics of tetrapod limbs. Reporter assays in zebrafish, where these limb regulatory elements are absent due to their origin in the lobe-finned lineage, still show their activity in fin development, demonstrating their conserved role across vertebrate evolution. Interestingly, we found more convergent dCNEs in caecilians and snakes, which have a longer evolutionary history of limblessness, compared to limbless lizards, which lost their limbs more recently. This pattern suggests that while some dCNEs may have contributed to limb loss, many likely accumulated due to relaxed natural selection over time. Therefore, this observation does not contradict prior studies suggesting varied mechanisms and evolutionary pathways leading to limblessness (Roscito et al. 2022). Furthermore, we found that despite relaxed selection, these dCNEs accumulate mutations more slowly than expected under neutral drift. This slower mutation rate may be due to pleiotropy, suggesting that these regulatory elements have roles in other developmental processes beyond limb formation. This finding underscores the complexity of regulatory element evolution and the potential for multiple selective pressures acting on these elements.

In conclusion, our findings reveal the complex genomic landscape underlying tetrapod limb loss and highlight the interplay between genes and regulatory elements in limb development. While our results suggest that regulatory changes play a dominant role in the evolution of limblessness, claiming that regulatory elements are universally more important than genes would be an oversimplification. Both components are indispensable: genes provide the essential proteins for limb formation, while regulatory elements orchestrate their precise spatial and temporal expression. The rare but impactful loss of critical genes like *Grem1* and *Tulp3* in some limbless lineages underscores the continued importance of coding sequences. Nevertheless, our study indicates that evolutionary changes leading to limb loss primarily involve modifications to regulatory elements. Many regulatory elements lack sequence conservation, suggesting that limbless tetrapods may harbor additional, unobserved regulatory changes. This aligns with increasing recognition in evolutionary developmental biology of the crucial role of gene regulation in morphological evolution. Future research should focus on elucidating the specific contributions of individual genes and regulatory elements to limb development and loss through functional studies and fine-scale comparative genomics across species with diverse limb morphologies.

## Supplementary Material

msae239_Supplementary_Data

## Data Availability

All sequencing data and genome assemblies have been deposited in the NCBI database under the accession number PRJNA1020273. All other study data are included in the article and/or supporting information.
