# Supplementary material for: Convergent Degenerated Regulatory Elements Associated with Limb Loss in Limbless Amphibians and Reptiles
Source: Mol Biol Evol. 2024 Nov 12;41(11):msae239. doi: 10.1093/molbev/msae239 (PMC11600591; doi:10.1093/molbev/msae239)
Supplement: msae239_Supplementary_Data [file msae239_supplementary_data.zip › Supplementary Information.pdf]

# Supplementary Information for
## Convergent degenerated regulatory elements associated with limb loss in limbless amphibians and reptiles

Chenglong Zhu[a,1], Shengyou Li[b,1], Daizhen Zhang[c,1], Jinjin Zhang[d,1], Gang Wang[c,1], Botong Zhou[a,1], Jiangmin Zheng[a], Wenjie Xu[a], Zhengfei Wang[c], Xueli Gao[a], Qiuning Liu[c], Tingfeng Xue[a], Huabin Zhang[c], Chunhui Li[a], Baoming Ge[c], Yuxuan Liu[a], Qiang Qiu[a], Huixian Zhang[e,2], Jinghui Huang[b,2], Boping Tang[c,2], and Kun Wang[a,2]

[a] Shaanxi Key Laboratory of Qinling Ecological Intelligent Monitoring and Protection, School of Ecology and Environment, Northwestern Polytechnical University, Xi'an 710072, China.
[b] Department of Orthopaedics, Xijing Hospital, The Fourth Military Medical University, Xi'an 710032, China
[c] Jiangsu Key Laboratory for Bioresources of Saline Soils, Jiangsu Provincial Key Laboratory of Coastal Wetland Bioresources and Environmental Protection, Jiangsu Synthetic Innovation Center for Coastal Bio-agriculture, Yancheng Teachers University, Yancheng City, 224002, China
[d] State Key Laboratory of Genetic Resources and Evolution, Kunming Natural History Museum of Zoology, Kunming Institute of Zoology, Chinese Academy of Sciences, Kunming, Yunnan, China
e CAS Key Laboratory of Tropical Marine Bio-Resources and Ecology, Guangdong Provincial Key Laboratory of Applied Marine Biology, South China Sea Institute of Oceanology, Chinese Academy of Sciences, Guangzhou, 510301, China

[1] C.Z., S.L., D.Z., J.Z., G.W., and B.Z. contributed equally to this work.
[2] To whom correspondence may be addressed. Email: wangkun@nwpu.edu.cn (K.W.); boptang@163.com (B.T.), huangjh@fmmu.edu.cn (J.H.), zhanghuixian@scsio.ac.cn (H.Z.)

**This PDF file includes:**

Figures S1 to S12
Legends for Tables S1 to S15

**Other supporting materials for this manuscript include the following:**

Supplementary Tables

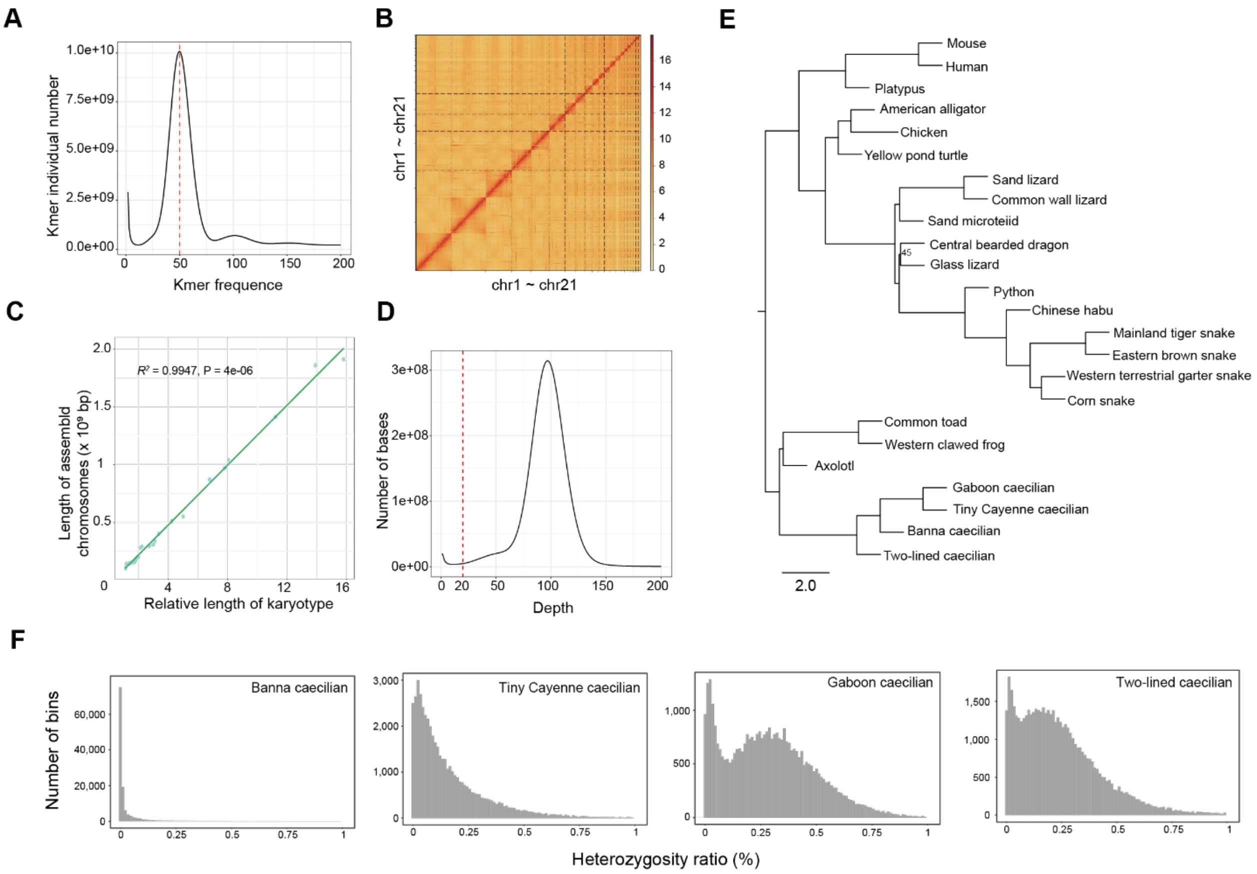


**Figure S1. Genomic characterization and phylogenetic relationships of Banna caecilian.**

(A) K-mer frequency distribution based on short reads. The estimate genome size based on this is 12.5 Gb. (B) Hi-C linkage density heat map of the Banna caecilian chromosome assembly. A total of 21 chromosomes were assembled. (C) The lengths of the assembled chromosomes match the relative lengths observed in previous karyotype studies. (D) The reads depth distribution for each site using short reads and long reads. The peak depth is 97 and 99% of bases are supported by more than 20 reads. (E) Species tree constructed by ASTRAL based on a set of bidirectional best-hit orthologous genes. All nodes are 100% supported except for the node of glass lizard and central bearded dragon, which is 45%. (F) Heterozygosity ratio per 100 kb in different individuals of Banna caecilian, tiny Cayenne caecilian, Gaboon caecilian and two-lined caecilian.

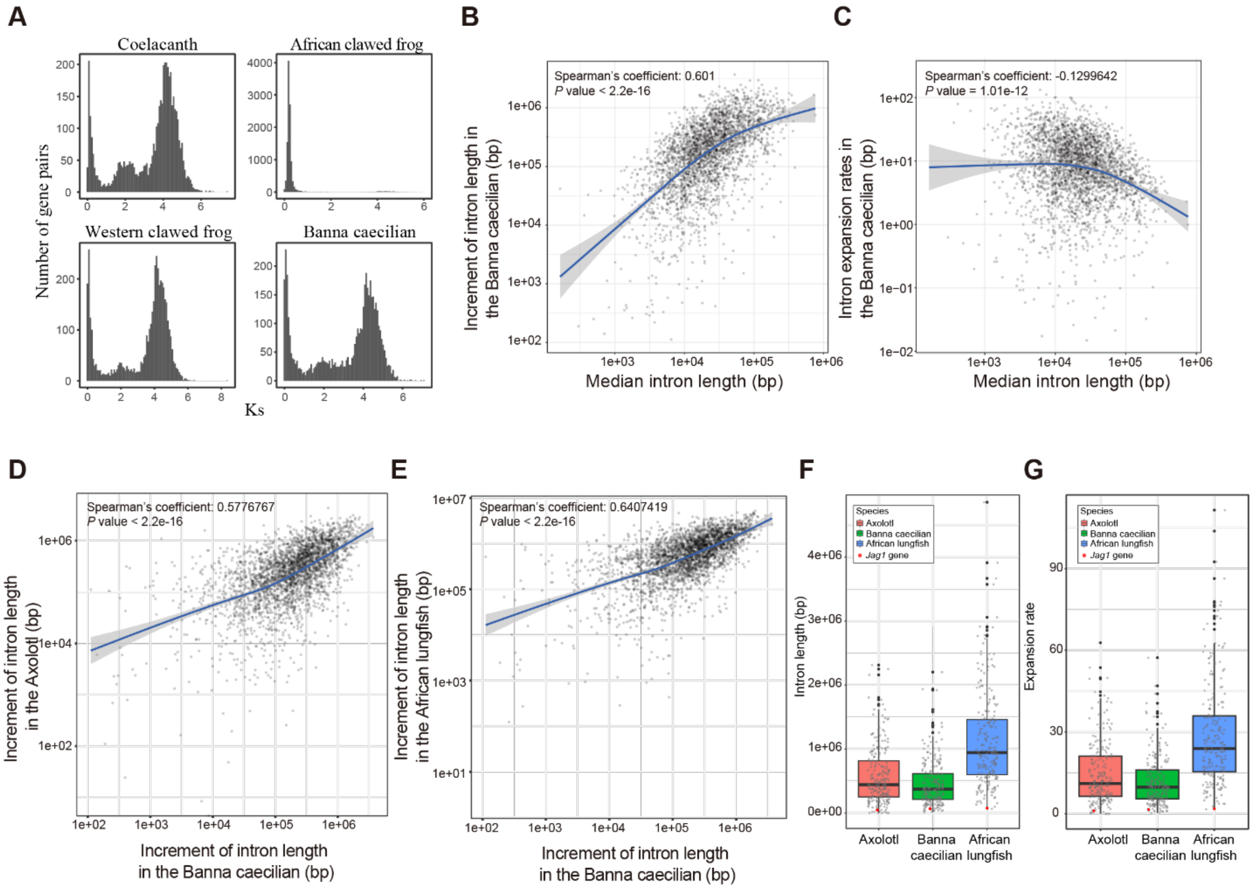


**Figure S2. The expansion of the Banna caecilian genome and the limited expansion of the *Jag1* gene.**

(A) Synonymous mutation rate (*Ks*) distribution between paralogous of the coelacanth, African clawed frog, Western clawed frog, and Banna caecilian. Among them, the African clawed frog is a species that recently underwent whole-genome duplication, while the other three species did not experience a recent whole-genome duplication event. (B) The relationship between median intron length and the increase in intron length in Banna caecilian. (C) The relationship between median intron length and the expansion rate of intron length in Banna caecilian. (D) The expanded intron size of Banna caecilian is positively correlated with the increment of intron length in the axolotl. (E) The expanded intron size of Banna caecilian is positively correlated with the increment of intron length in the African lungfish. (F) The intron length of the *Jag1* gene is short in large genomes compared to other genes with similar median intron length. (G) The intron length expansion rate of the *Jag1* gene is lower in large genomes compared to other genes with similar median intron length.

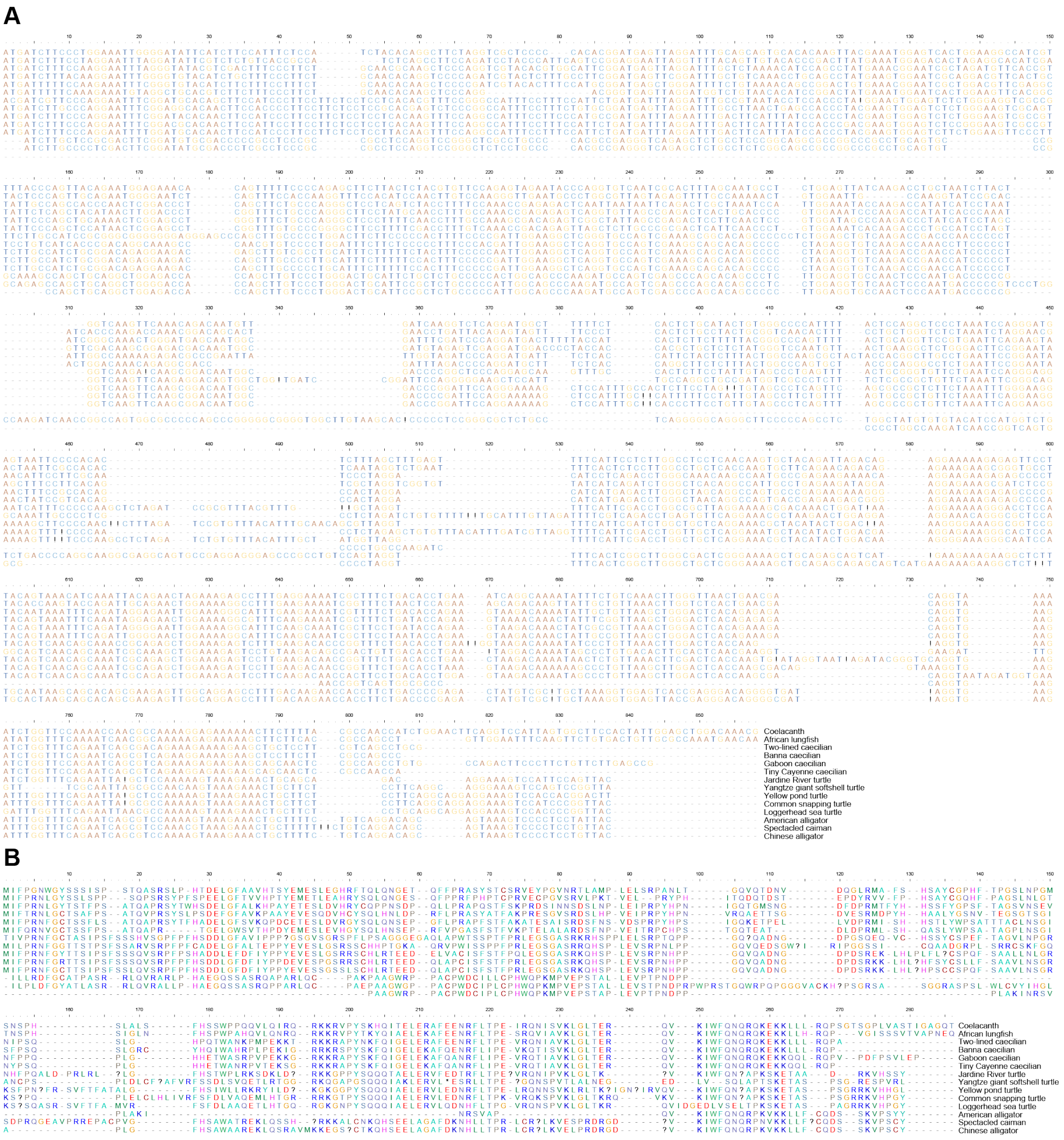


**Figure S3. *Hoxa14* exon sequence alignment and corresponding peptide alignment.**

(A) Codon alignment results of the *Hoxa14* exon sequence. (B) Alignment results of the corresponding amino acid sequences. "!"and "?"represent frameshift mutation sites, "*" represents premature stop sites.

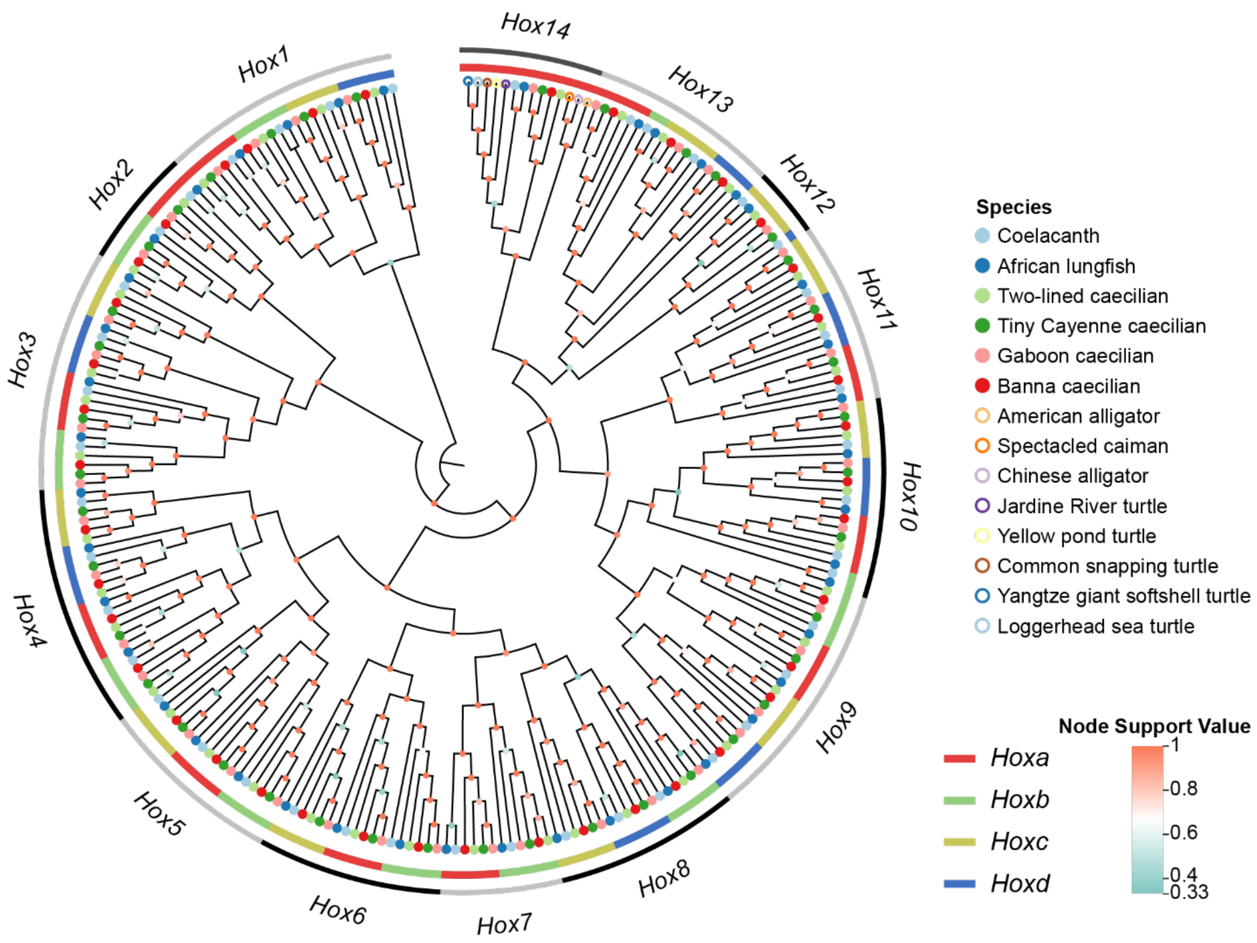


**Figure S4. The reconstructed phylogenetic tree of *Hox* cluster genes shows the retention of *Hoxa14* in caecilians.**

The reconstructed phylogenetic tree of *Hox* genes of caecilians, coelacanth and African lungfish and pseudo-*Hoxa14* genes of turtles and crocodilians. All *Hoxa14* genes are cluster in the one clade.

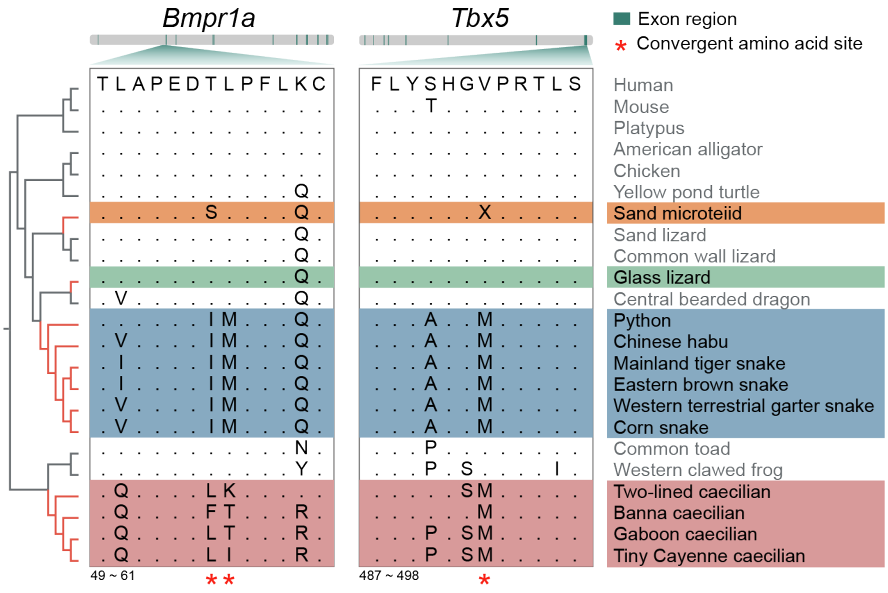


**Figure S5. Site-specific amino acid mutations in caecilians and snakes.**

Two cases of site-specific mutations shared between caecilians and snakes. The phylogenetic relationship is shown on the left side, and above is the gene structure.

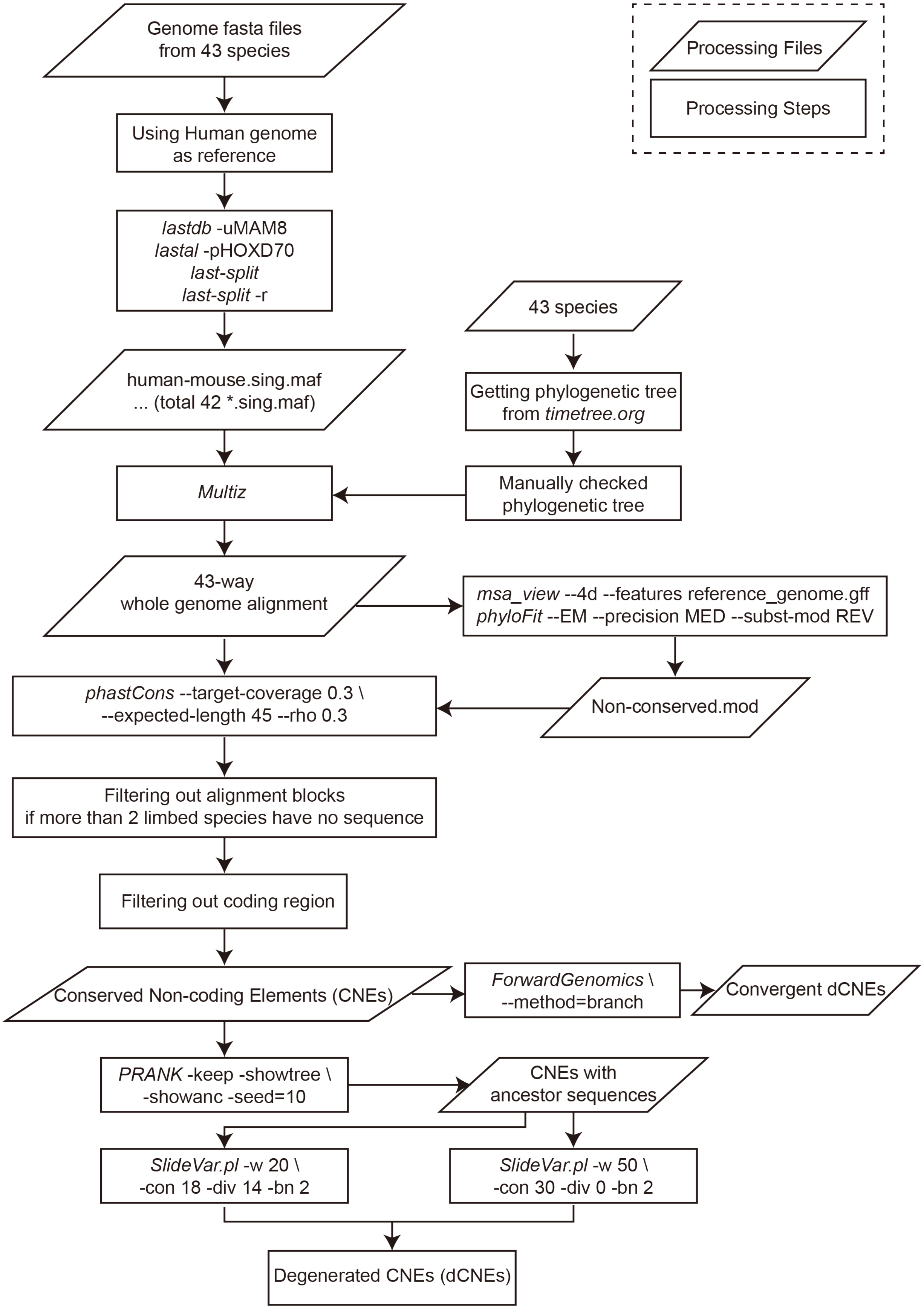
Genome fasta files
from 43 species
Processing Files
Processing Steps
Using Human genome
as reference
lastdb -uMAM8
lastal -pHOXD70
last-split
last-split -r
43 species
human-mouse.sing.maf
... (total 42 *.sing.maf)
Getting phylogenetic tree
from timetree.org
Multiz
Manually checked
phylogenetic tree
43-way
whole genome alignment
msa_view --4d --features reference_genome.gff
phyloFit --EM --precision MED --subst-mod REV
phastCons --target-coverage 0.3 \
--expected-length 45 --rho 0.3
Non-conserved.mod
Filtering out alignment blocks
if more than 2 limbed species have no sequence
Filtering out coding region
Conserved Non-coding Elements (CNEs)
ForwardGenomics \
--method=branch
Convergent dCNEs
PRANK -keep -showtree \
-showanc -seed=10
CNEs with
ancestor sequences
SlideVar.pl -w 20 \
-con 18 -div 14 -bn 2
SlideVar.pl -w 50 \
-con 30 -div 0 -bn 2
Degenerated CNEs (dCNEs)

**Figure S6. A step-by-step analysis pipeline for identifying CNEs and dCNEs.**

A step-by-step analysis pipeline for identifying CNEs and degenerated CNEs (dCNEs). In the flowchart, parallelograms represent input or generated files, while rectangles indicate analyses that need to be performed. All program names are italicized, and all other parameters not shown are default parameters. The "\" symbol is used as a connector between lines to indicate continuation.

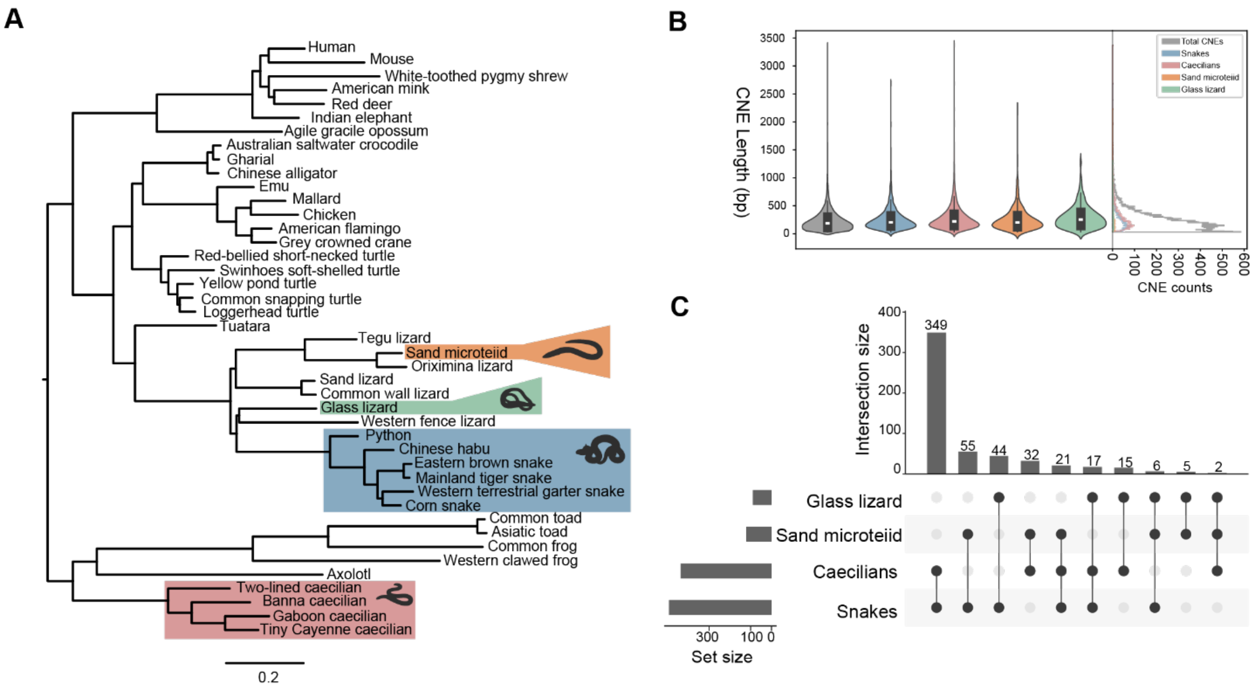


**Figure S7. Phylogenetic tree used for CNE analysis, statistical information of CNEs and limb-related convergent dCNEs.**

(A) The phylogenetic tree of the 43 tetrapods used in the CNE analysis. Branch lengths represent mutation rates calculated by phyloFit using 4-fold degenerate sites. (B) Length distribution of CNEs identified in tetrapods and diverged CNEs identified in different limbless lineages. The left panel shows a violin plot of CNE lengths, with the vertical line indicating the range from minimum to maximum length. The white horizontal line represents the median, while the black box denotes the interquartile range (25th to 75th percentiles). The right panel displays a histogram of CNE length frequencies, providing a detailed view of the distribution. (C) The number of limb enhancer signals in convergent dCNEs between different lineages.

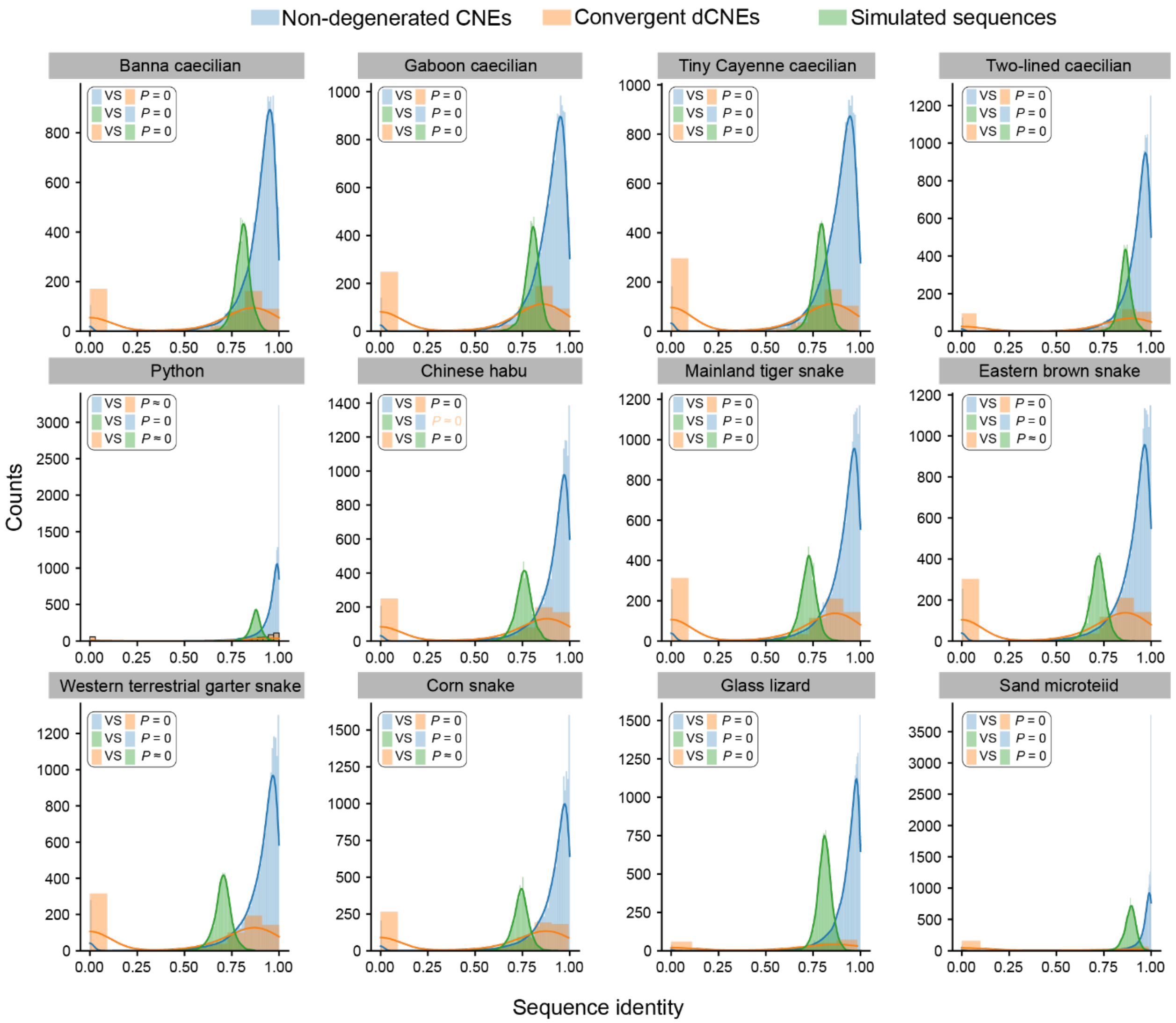


**Figure S8. The histogram distributions of non-degenerated CNEs, convergent dCNEs and simulations with a neutral substitution rate over the same period (Simulated sequences).**

The histogram distributions of non-degenerated CNEs, convergent dCNEs and simulations with a neutral substitution rate over the same period (Simulated sequences). The results of the t-tests indicate that there are significant differences between each pair of groups.

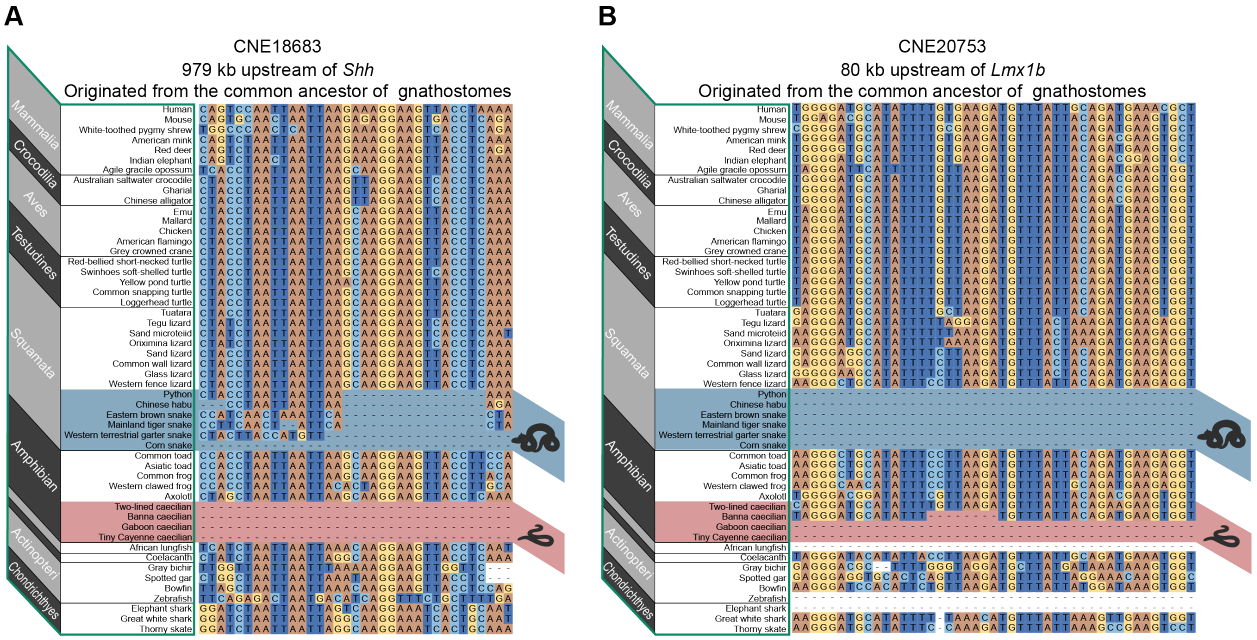


**Figure S9. Partial sequence alignment of CNE18683 and CNE20753.**

(A) Partial sequence alignment of CNE18683, which is located in ZRS enhancer and has degenerated in snakes and lost in all caecilians. (B) Partial sequence alignment of CNE20753, which is located in LARM1 enhancer and lost in all snakes and two caecilians.

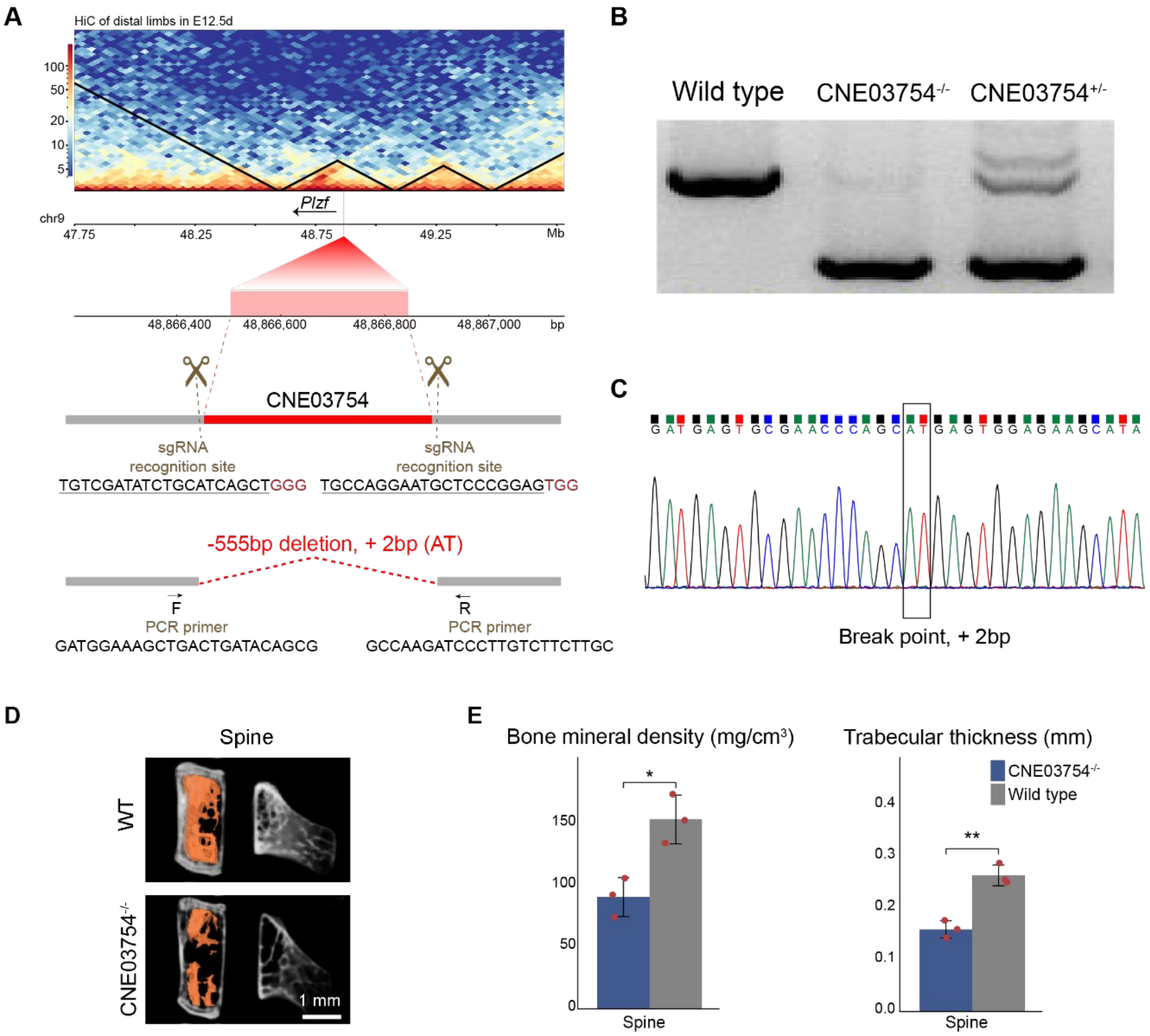


**Figure S10. CRISPR/Cas9 mediated the knockout of CNE03754.**

(A) Schematic diagram of knockout strategy. CNE03754 (chr9:48,866,503-48,866,845, mm10) and the closest gene *Plzf* were located in one TAD during mouse embryo development at 12.5 days in the distal limbs. Using CRISPR/Cas9, a 555bp deletion (chr9:48,866,340-48,866,894, mm10; another 2 bp insert: AT) knockout mouse was generated and used for further analysis. Genotyping primers are marked with black arrows (F and R). (B) PCR validation strategy and results for CNE03754 knockout mouse. (C) Sanger sequencing traces show the deletion break point of CNE03754$^{-/-}$ mice. (D) Micro-CT images of the spine in wild-type (WT) and CNE03754$^{-/-}$ mice (n = 3). Images captured shows the reduced bone density in spine. (E) Statistics of bone density and trabecular thickness of spine in WT and CNE03754$^{-/-}$ mice. The data is shown as the mean ± SD (standard deviation). **P* value < 0.05, ***P* value < 0.01.

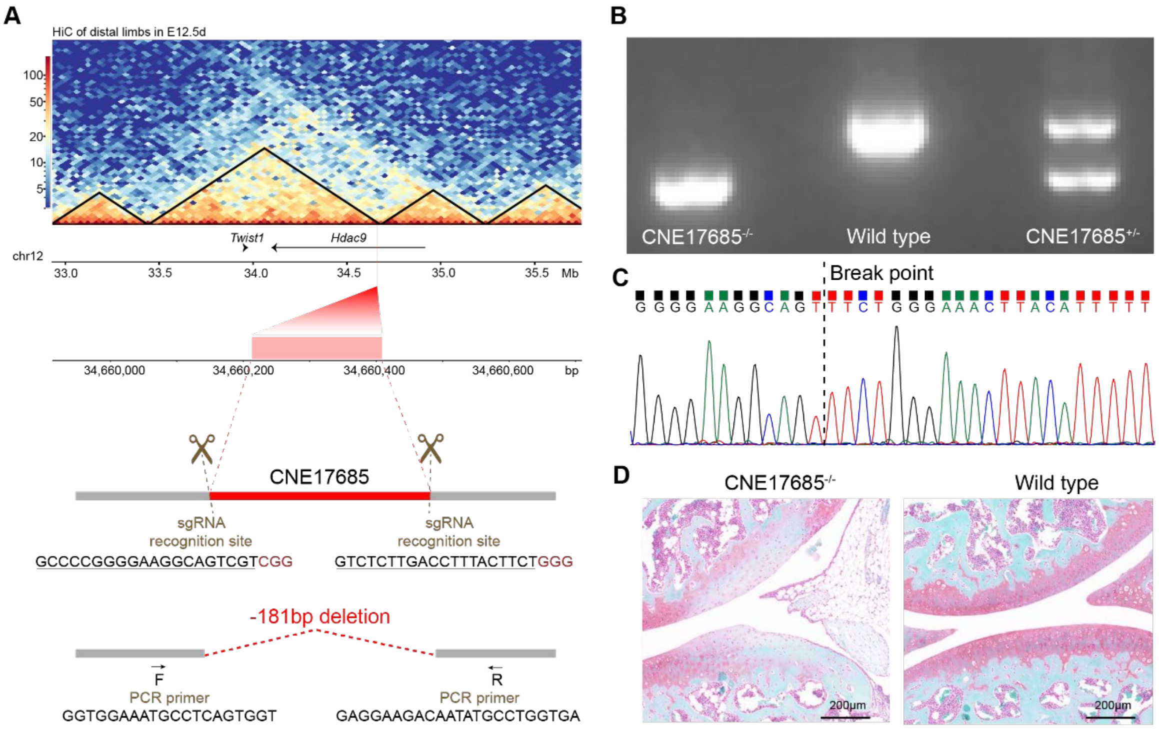


**Figure S11. CRISPR/Cas9 mediated the knockout of CNE17685.**

(A) Schematic diagram of knockout strategy. CNE17685 (chr12:34,660,214-34,660,410, mm10) and the gene *Twist1* were located in one TAD during mouse embryo development at 12.5 days in the distal limbs. Using CRISPR/Cas9, 181bp deletion (chr12:34,660,209-34,660,389, mm10) knockout mice were generated and used for further analysis. Genotyping primers are marked with black arrows (F and R). (B) PCR validation strategy and results for CNE17685 knockout mouse. (C) Sanger sequencing traces show the deletion break point of CNE17685$^{-/-}$ mice. (D) Safranin O-fast green staining of the knee joint indicates cartilage abnormalities in the CNE17685$^{-/-}$ knockout mice (n = 3).

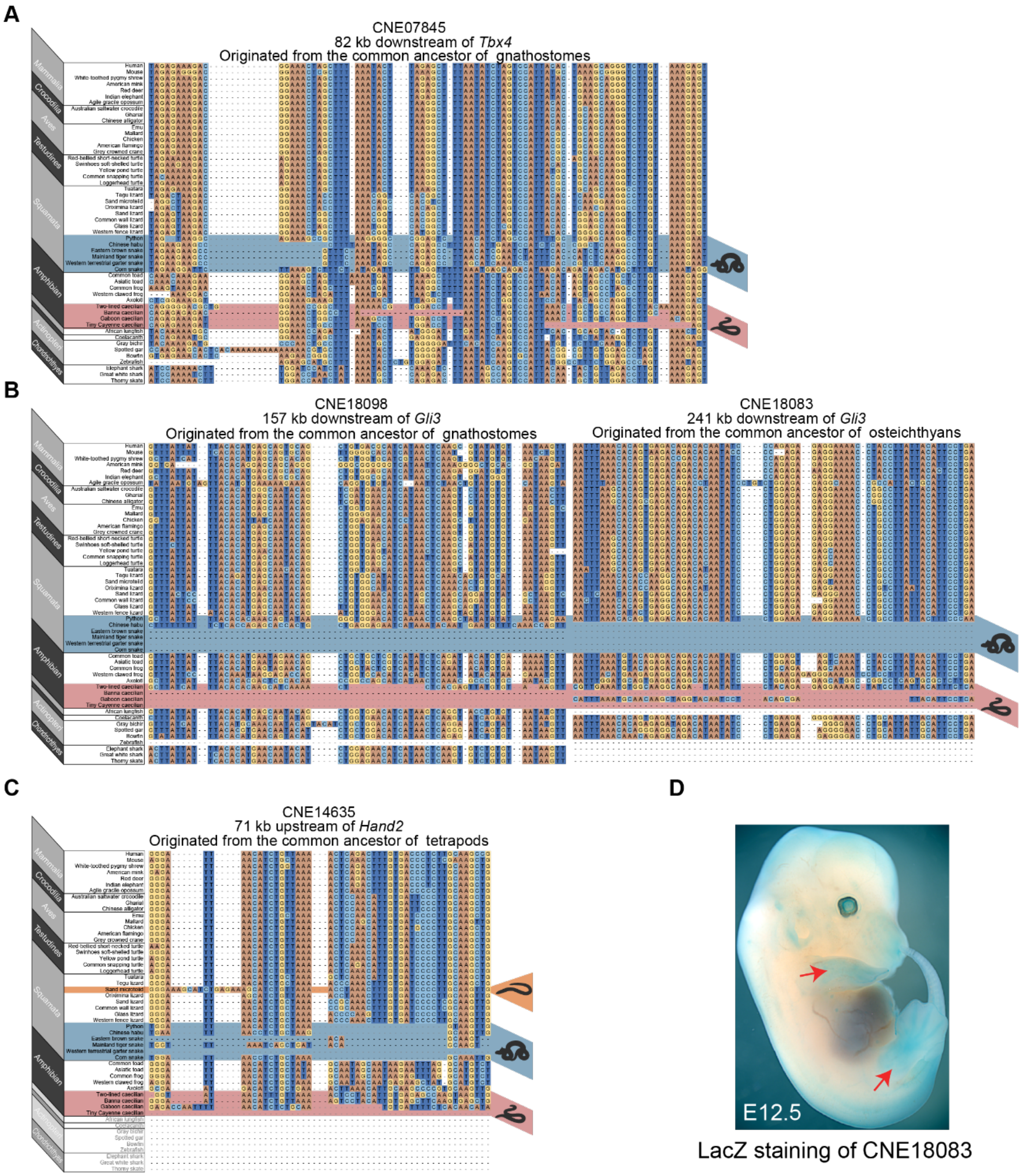


**Figure S12. Four convergent dCNEs may be important for limb development.**

(A) CNE07845 (chr17:61,534,462-61,534,808, hg38) is located 82 kb downstream of *Tbx4* and has an origin from the common ancestor of the gnathostomes. (B) CNE18098 (chr7:42,080,907-42,081,151, hg38) is located 157 kb downstream of *Gli3* and has an origin from the common ancestor of the gnathostomes. CNE18083 (chr7:41,996,381-41,996,591, hg38), located 241 kb downstream of *Gli3*, which origins from the common ancestor of osteichthyans, have lost in five snakes and two caecilians. (C) CNE14635 (chr4:173,601,533-173,601,761, hg38) is located 714 kb upstream of *Hand2* and has an origin from the common ancestor of the tetrapods. (D) The LacZ staining assay

demonstrates that CNE18083 exhibits enhancer activity in both the forelimbs and hindlimbs of mouse embryos at E12.5 (n = 3).

**Legends for Tables S1 to S15**

Table S1. Sequencing data statistics for Banna caecilian.
Table S2. Genome assembly statistics for Banna caecilian.
Table S3. BUSCO results for gene structure annotation in Banna caecilian genome.
Table S4. Reconstruction results for ancestral chromosomes of tetrapods.
Table S5. List of 49 genes with limited intron lengths in Banna caecilian, African lungfish, and axolotl.
Table S6. Gene Ontology enrichment results for the 49 genes with limited intron lengths.
Table S7. Reconstruction results for ancestral chromosomes in tetrapods.
Table S8. Limb-related gene used in this study.
Table S9. Gene list of amino acid mutations at the same site in snakes and caecilians with at least 7 conserved amino acids around the site.
Table S10. H3K27ac ChIP-seq data from ENCODE database used in this study.
Table S11. Statistical results of convergent dCNEs, and rate of convergent dCNE in limbless tetrapods.
Table S12. Convergent dCNEs identified from the Forward Genomics software.
Table S13. Genomic location, origin information and H3K27ac enhancer signal statistics for 1,251 convergent dCNEs.
Table S14. Statistical results of CNE03754 knockout and wild-type mice for bone mineral density and trabecular thickness.
Table S15. Sources of genomic data for all species used in this study.

**Supplementary Tables (separate file).** All supplementary tables from Table S1 to Table S15.
